# TGFβ1-RCN3-TGFBR1 loop facilitates pulmonary fibrosis by orchestrating fibroblast activation

**DOI:** 10.1186/s12931-023-02533-z

**Published:** 2023-09-14

**Authors:** Mingting Wu, Zhenyan Wang, Xiaoqian Shi, Danni Zan, Hong Chen, Shuqiao Yang, Fangping Ding, Liu Yang, Pingping Tan, Runlin Z. Ma, Jing Wang, Lishuang Ma, Yingmin Ma, Jiawei Jin

**Affiliations:** 1 Department of Respiratory and Critical Care Medicine, Beijing Institute of Respiratory Medicine and Beijing Chao-Yang Hospital Jingxi Campus, Capital Medical University, No. 5 Jingyuan Road, Beijing, China; 2 Medical Research Center, Beijing Institute of Respiratory Medicine and Beijing Chao-Yang Hospital, Capital Medical University, Beijing, China; 3 Department of Pathology, Beijing Chao-Yang Hospital, Capital Medical University, Beijing, China; 4 Department of Respiratory and Critical Care Medicine, Beijing Institute of Hepatology, Beijing Youan Hospital, Capital Medical University, No. 8, Xi Tou Tiao, Youanmen Wai, Beijing, China; 5 State Key Laboratory of Molecular Developmental Biology, Institute of Genetics and Developmental Biology, Chinese Academy of Sciences, Beijing, China; 6 Department of Neonatal Surgery, Children’s Hospital of Capital Institute of Pediatrics, Peking Union Medical College, Beijing, China

**Keywords:** Reticulocalbin 3, Pulmonary fibrosis, Fibroblasts, TGFβ1, TGFβ1 receptor type 1 (TGFBR1), Enhancer of zeste homolog 2 (EZH2)

## Abstract

**Background:**

Idiopathic pulmonary fibrosis (IPF) bears high mortality due to unclear pathogenesis and limited therapeutic options. Therefore, identifying novel regulators is required to develop alternative therapeutic strategies.

**Methods:**

The lung fibroblasts from IPF patients and Reticulocalbin 3 (RCN3) fibroblast-selective knockdown mouse model were used to determine the importance of Rcn3 in IPF; the epigenetic analysis and protein interaction assays, including BioID, were used for mechanistic studies.

**Results:**

Reticulocalbin 3 (RCN3) upregulation is associated with the fibrotic activation of lung fibroblasts from IPF patients and Rcn3 overexpression blunts the antifibrotic effects of pirfenidone and nintedanib. Moreover, repressing Rcn3 expression in mouse fibroblasts ameliorates bleomycin-induced lung fibrosis and pulmonary dysfunction in vivo. Mechanistically, RCN3 promotes fibroblast activation by maintaining persistent activation of TGFβ1 signalling via the TGFβ1-RCN3-TGFBR1 positive feedback loop, in which RCN3 upregulated by TGFβ1 exposure detains EZH2 (an epigenetic methyltransferase) in the cytoplasm through RCN3-EZH2 interaction, leading to the release of the EZH2-H3K27me3 epigenetic repression of TGFBR1 and the persistent expression of TGFBR1.

**Conclusions:**

These findings introduce a novel regulating mechanism of TGFβ1 signalling in fibroblasts and uncover a critical role of the RCN3-mediated loop in lung fibrosis. RCN3 upregulation may cause resistance to IPF treatment and targeting RCN3 could be a novel approach to ameliorate pulmonary fibrosis.

**Graphical Abstract:**

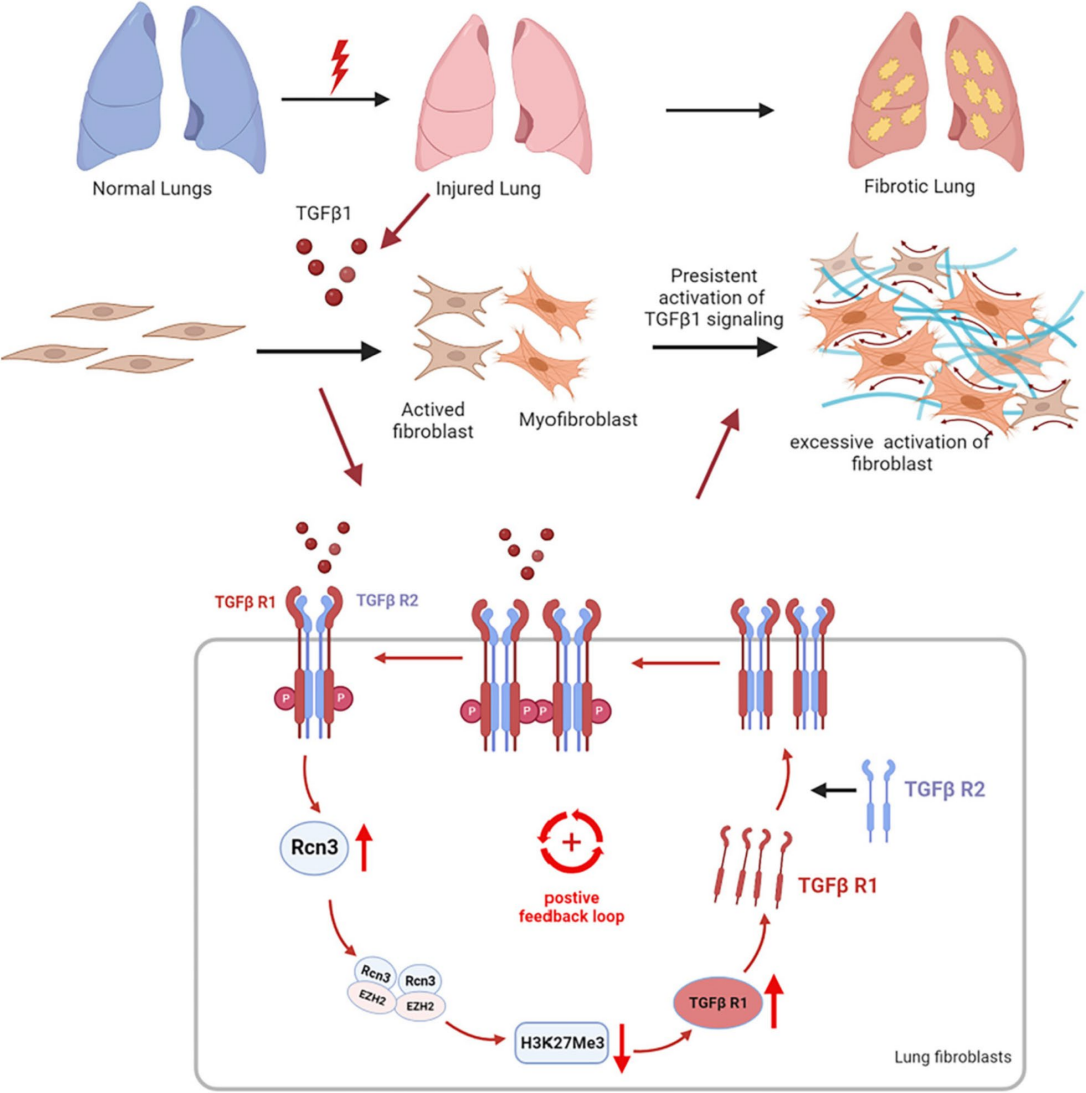

**Supplementary Information:**

The online version contains supplementary material available at 10.1186/s12931-023-02533-z.

## Background

Idiopathic pulmonary fibrosis (IPF) is the most aggressive fibrotic intestinal lung disease (ILD) with irreversible destruction of lung architecture, and it bears high mortality due to unclear pathogenesis and limited therapeutic options [1, 2]. The inevitable progression of IPF is linked to its complex pathogenesis, which involves with different cell types, signalling pathways, and structural changes [3]. Despite past extensive studies, the mortality of IPF is barely improved [1, 2]. IPF is believed to arise from aberrant fibrotic remodelling caused by excessive fibroblast activation, which is associated with the deregulation of signalling initiated by fibrotic factors, such as transforming growth factor-β (TGF-β), fibroblast growth factor (FGF) and platelet-derived growth factor (PDGF) [1]. Recently, two approved antifibrotic drugs (pirfenidone and nintedanib) targeting these signalling pathways can slow the progression and relieve symptoms in IPF patients. However, they still exhibit inconsistent efficacy and fail to reduce mortality [3–6]. Therefore, identifying novel regulators is required to develop alternative therapeutic strategies.

Repeated injuries to the alveolar epithelium are primarily cause of IPF, resulting in the release of profibrotic mediators. The most predominant mediator is TGFβ1 which induces fibroblast activation through both canonical and non-canonical pathways, including smad, AKT, ERK and STAT3 pathways [7–9]. TGFβ1 binds to the type II receptor (TGFBR2) and then recruits the type I receptor (TGFBR1) to the heterotetrametric complex, activating TGFBR1 which then phosphorylates and activates downstream effectors [10]. The regulation of TGFBRs through transcription, protein stability and trafficking are essential for maintaining TGF-β signalling balance [11, 12]. Increasing evidence indicates that the epigenetic regulation of TGFBRs also plays a critical role in the dysregulation of TGF-β signalling in cancers [13–16]. However, the regulatory mechanism in the upstream of TGFβ1 signalling during pulmonary fibrosis remains unclear.

Reticulocalbin 3 (RCN3), as an ER chaperone protein localised to the secretory pathway, contains a signal sequence at its N-terminus, six EF-hands, and an HDEL ER-retention signal at its C-terminus [17–19]. Our earlier study showed that Rcn3 deletion caused a failure of alveolar epithelial cells (AECII) maturation with the impaired secretion of surfactant proteins, leading to disrupted perinatal lung development [19]. In addition, our recent studies indicated the critical roles of Rcn3 in regulating AEC apoptosis and inflammatory response, involving in lung fibrosis and acute lung injury (ALI) [20, 21]. Interestingly, we found a manifest upregulation of Rcn3 in the fibrotic area of the lung from the bleomycin-induced fibrosis mouse model, suggesting a potential role of Rcn3 in fibroblast during pulmonary fibrosis [21]. Increasing studies also showed that Rcn3 was associated with collagen production and collagen fibrillogenesis in cardiac fibrosis and postnatal tendon development [22, 23]. Given these findings, we hypothesised that RCN3 could be a critical regulator in modulating fibroblast activation during pulmonary fibrosis.

Herein, we found that RCN3 was remarkably upregulated in fibroblasts from both patient and mouse fibrotic lungs, whereas RCN3 knockdown diminished the activation of disease human lung fibroblasts from IPF patients (DHLF-IPF). On the other hand, Rcn3 overexpression induced fibroblast activation and significantly blunted the anti-fibrotic effects of both pirfenidone and nintedanib. Furthermore, fibroblast-selective Rcn3 in vivo knockdown ameliorated bleomycin-induced lung fibrosis and pulmonary dysfunction. The mechanistic study revealed that RCN3 promoted fibrogenesis by enhancing TGFβ1 signalling via the TGFβ1-RCN3-TGFBR1 positive feedback loop. This loop epigenetically maintains TGFBR1 transcription by releasing the EZH2-H3K27me3 epigenetic repression via the Rcn3-EZH2 interaction, leading to persistent activation of TGFβ1 signalling. These findings suggest a determinate role of the Rcn3-mediated loop in lung fibrosis and introduce a novel regulating mechanism for TGFβ1 signalling. Elevated RCN3 level may contribute to resistance to IPF treatment, so targeting Rcn3 could be a novel therapeutic approach for pulmonary fibrosis.

## Methods

### Mouse models

All animal procedures were approved by the Animal Care and Ethics Committee of the Institute of Genetics and Developmental Biology, Chinese Academy of Sciences and were performed in accordance with the Guide for the Care and Use of Laboratory Animals of the Chinese Academy of Sciences. By crossing the fibroblast-specific protein (FSP)1-Cre (Jackson Lab) and Rcn3^flox/flox^ mice (generated in our lab [21]), the fibroblast-selective *Rcn3* deletion mice (CKO, FSP1-Cre/Rcn3^f/f^) were generated and the littermates (Rcn3^flox/flox^) served as controls. The intratracheal administration of bleomycin (Sigma-Aldrich) was performed in 8-week-old mice at the dose of 0.08 U/kg in 50 μl saline (25 μl × 2), and sterile saline was instilled at the same procedure as a control [21].

### Human tissues

Human fibrotic lung samples were obtained by diagnostic surgical lung biopsies from patients diagnosed as IPF at Beijing Chao-Yang Hospital. Normal human lung samples were collected from surgical lung resections from patients with lung cancer at the same hospital. All studies involving human lung tissues were approved by the Ethics Committee of Beijing Chao-Yang Hospital (2021-KE-295) and signed informed consents for research use of samples were obtained from all subjects. All methods were carried out in accordance with relevant guidelines and regulations.

### Measurement of airway resistance and compliance

The airway resistance and compliance were measured using AniRes 2005 system (Beijing Biolab, Beijing, China) as manufacturer’s manual and previously described [24]. Briefly, mice were anesthetised by intraperitoneal injection of avertin (250 mg/kg) followed by tracheal exposure and cannulation with a Y-type cannula (2.5 mm inner diameter). After the mice were then placed inside a plethysmographic chamber at the supine position, one branch of the tracheal Y-type cannula was connected to a ventilator at outside of the chamber and the other was connected to the pressure detection channel. The mice were mechanically ventilated at a breath rate of 90 breaths/minute at a tidal volume equal to 10 ml/kg with maintaining peak respiratory pressure at 10–16 cm of water. The display pulmonary function parameters were calculated, including forced vital capacity (FVC), dynamic compliance (Cdyn), inspiratory resistance (Ri), and expiratory resistance (Re).

### Histological and immunohistochemical staining

The lung tissues fixed with 4% paraformaldehyde (PFA) were embedded in paraffin and sliced into 5 µm thickness for Haematoxylin & eosin (HE) and Masson’s Trichrome staining (ScyTek Laboratories). The fibrotic score for mouse fibrotic lung was scored as semi-quantitative (0–4 score), as previously described [21]. Through evaluating 10 sequential and nonoverlapping fields on Masson’s trichrome-stained sections for each mouse, the fibrotic condition was scored: 0, normal lung architecture; 1, increased thickness of some (≤ 50%) of interalveolar septa; 2, thickening of > 50% of interalveolar septa without formation of fibrotic patches; 3, thickening of the interalveolar septa with formation of isolated fibrotic patches; 4, formation of multiple fibrotic patches with total or subtotal distortion of parenchymal architecture.

For immunohistochemical staining, paraffin-embedded tissues were performed through standard methods and incubated with primary antibodies: anti-αSMA (1:2000, Abcam: ab124964) and anti-Rcn3 (1:4000, sigma: HPA050402).

### The isolation of mouse alveolar epithelial cells (AECs) and lung fibroblasts

Mouse alveolar epithelial cells were prepared as described in our previous published study [21]. Briefly, the trachea in exsanguinated mice were cannulated and 2 ml of 5 U/ml dispase II (Sigma, D4693) in DPBS was rapidly instilled into the lung, followed by a slow infusion of 0.5 ml agarose (45 °C 1% w/v, low melting). The lung was taken out and incubated in dispase for 45 min (25 °C), and then lung tissue was gently teased from the bronchi in HEPES-buffered DMEM containing 100 U/ml DNase I. The cell suspension was filtered through progressively smaller cell strainers (100 and 40 μm) and metal gauze (25 μm), and cells were placed on CD 45/32 coated culture dished for 1 h. Afterwards, fibroblasts were enriched by adherence for 2 h on cell culture dishes loosely. The adhered fibroblast and unadhered AECII cells were collected to check Rcn3 expression.

The mouse lung fibroblasts were prepared as below. Mouse lungs perfused by DPBS were harvested, minced and digested in HBSS buffer containing 0.1% type I collagenase, 2.5 U/ml dispase II and 100 U/ml DNase I for 1 h at 37 °C water bath. The digestion was mixed with DMEM (10% FBS), followed by pipetting to separate cells from tissues. The cells were plated into cell culture dishes and incubated at 37 °C for 24 h, followed by washing and cultured for further experiments.

### Human lung fibroblasts (HLF)

Human lung fibroblasts were purchased from merchandise. Normal primary human lung fibroblasts (NHLF) and Disease human lung fibroblasts derived from IPF patients (DHLF-IPF) were purchased from Lonza (American, CC-2512 and CC-7231). Cells were cultured in Fibroblast Medium (CM-H011, Procell) and DMEM (sigma, D6429).

Human lung fibroblasts were prepared from human lung tissues. Fresh lung tissue was cut into small pieces and washed with DPBS three or more times till clean blood. The lung tissues were further cut into smaller fragments (~ 1 mm^3^), pooled in DMEM (10% FBS) and plated into cell culture dishes. After fibroblasts proliferated from these tissues, the tissue pieces were washed out. The fibroblast finally grew to 80% confluence and serially passed in the same medium.

### Cell fraction isolation

Isolated different cell protein components (cytoplasmic, nuclear and membrane proteins) using a protein extraction kit (KeyGen BioTEC, KGBSP002) following the product manufactures instruction. The Histone protein was extracted using EpiQuik™ Total Histone Extraction Kit (EPIGENTEC, OP-0006) as manufacturer’s manual. Briefly, resuspend cells in diluted pre-lysis buffer on ice for 10 min with gentle shaking. After the supernatant was removed by centrifugation at 12000×*g*, the pellet was mixed with lysis buffer for 30 min on ice. The supernatant containing histone was collected by spinning at 12,000 rpm and mixed with 0.3 volume of balance-DTT buffer immediately for the following experiments.

### RCN3 knockdown and over-expression

NHLF were planted about 2 × 10^5^ cells per well in six wells plates with 10% FBS DMEM overnight, and cells were transfected by 50 uM of siRNA-RCN3 (targeting sequence shown below) or siRNA-scramble through lipo3000 (Invitrogen, L3000015) in Opti-MEM (Gibco, 51985034). 36 h post transfection, cells were cultured in FBS free medium overnight and then treated with or without TGFβ1 (sigma, GF346) at 5 ng/ml for 24 h (for qPCR) or 48 h (for Western blot). As for Rcn3 overexpression, the lentiviral particles containing human *Rcn3* (NM_020650.3) or empty vector were produced in 293T cells using lentiviral shuttle vector FUGW. 24 h after infection, cells were subjected to further experiments. The siRNA sequence for human Rcn3:

- RNA oligo sequences 21nt guide UUCAGCAAUCACGAUGUCCCG;
- 21nt passenger GGACAUCGUGAUUGCUGAAAC.

### TGFβ1 stimulation and inhibition in fibroblasts

HLF and MLF were cultured in serum-free medium and treated with TGFβ1 (sigma, GF346) at 5 ng/ml or other concentrations as indicated. For inhibition, the cells were co-treated by inhibitors 4-PBA (MCE, HY-A0281) or LY2109761 (sigma, HY-12075-TMP) with TGFβ1, followed by downstream experiments. For assaying signalling activation in response to TGFβ1 stimulation, after 5 ng/ml of TGFβ1 treatment, cell lysates were collected at 30 min for p-smad3 and at 12 h for p-AKT and p-stat3.

### Bioi-ID assay and protein–protein interactions

The BioID-Rcn3 fusion protein was constructed by inserting *Rcn3* (without signal peptide) into the C-terminal of BioID2 and the signal peptide fragment of Rcn3 into the N-terminal of BioID2; BioID-only was constructed by inserting Rcn3 signal peptide segment in the N-terminal of BioID2. The overexpression of BioID-Rcn3 and BioID-only by inserting into lentiviral shuttle vector FUGW and the lentiviral were produced to infect normal human lung fibroblast (NHLF). After the expressions of these proteins were validated, the BioID assay was performed according to the previous detailed protocol [25]. Briefly, cells reached approximately 70–80% confluency in 100 mm dishes and were infected by lentivirus for 24 h, followed by exposure to TGFβ1 (5 ng/ml) and biotin (50 µM, sigma, B4639) for another 24 h. The cell lysate was triturated on ice by sonication twice and subjected into streptavidin-affinity purification process to enrich biotinylated proteins proximal to Rcn3. The enriched proteins were identified by LC–MS/MS. By eliminating the common proteins in BioID-Rcn3, BioID-only and Biotin controls, specific potential interaction proteins with Rcn3 were identified.

### Quantitative real-time PCR (qPCR)

RNA was isolated from cells or tissues with Trizol (Invitrogen, 15596018) according to the standard protocols, and the cDNA was synthesised using PrimeSTAR® Max DNA Polymerase (Takara, R045A). qPCR assays on these cDNA were performed using TB Green® Premix Ex Taq™ II (Takara, RR820) and data were normalised to house-keeping gene (*RPL19* content for mouse samples and *GAPDH* for human samples) and analysed by the 2^−△△^Ct method relative to saline/vehicle treated control groups. The data are derived from at least three independent experiments performed in triplicate and the primers used in qPCR see Additional file 2: Table S1.

### Biolayer interferometry (BLI)

Rcn3–EZH2 direct interaction determined by biolayer interferometry technology by OctetRED system (ForteBio) using human recombinant proteins (hrRcn3: Abcam, ab276552; EZH2: Origene, TP302054), following manufacturer’s protocol. In brief, rhEZH2 was biotinylated by Biotinylation Kit (Thermo Scientific, 21329) followed by desalting using desalt columns (thermos scientific, 89889). rhRcn3 with his-tag (5 µg/ml) were immobilised with NTA biosensors (ForteBio, 18-5001) followed by washing with binding buffer three times. The biotinylated rhEZH2 was serially diluted in binding buffer (262.3, 131.1, 65.56 and 32.78 µg/ml) and the Rcn3-EZH2 association and dissociation were detected by OctetRED system for 10 min at 25 °C. The baseline signal drift was controlled by monitoring immobilized Rcn3 without EZH2. OctetRED analysis software was used to analyse the data.

### Immunoblotting assay

30–50 µg of extracted protein containing protease inhibitor and phosphatase inhibitor was boiled in loading buffer and was separated by 8–12% SDS-PAGE, flowed by blotting onto 0.2 µm PVDF membrane. The membrane was then blocked with 5% skim-milk in Tris-buffered saline, 0.1% Tween 20 (TBS-T) for 90 min at room temperature and in turn incubated with diluent primary antibody as recommended in the instructions overnight at 4 °C. After washing with TBS-T, the membrane was incubated with horseradish peroxidase (HRP)-conjugated secondary antibody at room temperature and signals were visualised by enhanced chemiluminescence (GE Healthcare). The band-densitometry data were measured by using the Image J software. The antibodies were listed in Additional file 2: Table S2.

### Hydroxyproline assay

The lung tissue samples (right middle lobes) were transferred to 6 M HCl to final 100 mg/ml, followed by incubation for 20 h at 95 °C. After centrifugation at 13000×*g*, the supernatant was diluted with water to 4 M HCL at the ratio of 1 volume sample to 0.5 volume water and then 35 µl of the sample was used to quantitate collagen content by using a collagen assay kit (QuickZyme, QZBtotcol1-TMP) according to manufacturer’s manual.

### Cell proliferation assay

#### CCK8 assay

After transferring siRNA for 24 h, cells were seeded in a 96-well cell culture plate at a density of 5 × 10^3^ for 24 h followed by 12 h of serum starvation. Cells were then exposed to TGFβ1 (5 ng/ml), FGF (25 ng/ml, R&D Systems, 3718-FB-010) or 10% FBS. After 24 h post treatment, cell proliferation was detected by adding 10 µl of CCK8 solution for each well and incubating for 2 h in 37 °C (CCK8 assay kit, LABLEAD, CK001). The final absorbance at 450 nm was used to calculate cellular proliferation rate.

#### EDU proliferation assay

Cell proliferation was measured by EdU Cell Proliferation Kit with Alexa Fluor 488 (Beyotime, C0071S) according to the instruction. Briefly, after cells were treated with TGFβ1 (5 ng/ml) for 24 h, pre-heat EDU working solution (10 μM) was added into each well followed by 2 h of incubation at 37 °C. Cells were fixed in 4% PFA and permeabilizated with 0.3% Triton X-100 in DPBS. Cells were incubated with click addictive solution and stained with Hoechst 33342. The images of EDU were photographed by Leica fluorescence microscopy and calculated by Image J software.

### Cell migration assay

#### Transwell assay

siRNA transfected cells were reseeded with serum free medium at the density of 1 × 10^4^ into the upper chamber, while the lower chamber was equipped with DMEM containing 10% FBS. After 24 h, cells in the upper chamber were fixed by 4% PFA and stained by 0.1% Crystal Violet Ammonium Oxalate Solution (solarbio, G1063). Each chamber was photographed for 9 views under the inverted microscope and calculated the number of invaded cells through Image J software.

#### Scratch assay

Until the seeded transfected cells reached 90% on the 6-well plate, scrapped two straight lines on each well and washed with DPBS to remove detached cells. Cells were incubated with or without TGFβ1 (5 ng/ml) in 1% FBS DMEM for 48 h photographed and recorded the images at 0 and 48 h. Compared each image and obtained the distance of each scratch closure by Image J software.

### High-throughput RNA sequencing and data analyses

RNA-Sequencing analyses of the total RNA from NHLF cells with Rcn3-siRNA (Si-T) and Ctl-siRNA (SiC-T) after TGFβ1 exposure (5 ng/ml) for 24 h (n = 4 per group) by OE Biotech (Shanghai, China). In brief, total RNA was extracted using the mirVana miRNA Isolation Kit (Ambion) and The RNA Integrity Number (RIN) ≥ 7 were subjected to libraries construction using TruSeq Stranded mRNA LTSample Prep Kit (Illumina, San Diego, CA, USA). RNA sequencing was performed using the Illumina sequencing platform (HiSeqTM 2500 or Illumina HiSeq X Ten) and 125 bp/150 bp paired-end reads were generated. Quantification of mRNA transcript abundance was performed by normalised expression values as fragments per kb per million reads using cufflinks, and the read counts of each gene were obtained by htseq-count. A total of 180 differentially-expressed genes (DEGs) with the foldchange (FC) > 1.2 and FDR < 0.05 were identified between Si-T versus SiC-T by using DEseq. Hierarchical clustering presented the gene expression profiles separated based on *Rcn3* knockdown. The Gene Ontology analysis on these DEGs prioritised by strength (Gene-ratio: the ratio of number of deferentially expressed genes between the number of genes associated the GO term) and FDR p values corrected by BH procedure (< 0.05); The most affected processes were presented. BP: Biological process, CC: Cellular component, MF: Molecular function.

### Protein stability assay

Cycloheximide pulse chase assay was used for examining TGFBR1 protein stability. Control and Rcn3-knockdown NHLF were treated with 100 μg/ml cycloheximide (MedChemExpress, HY-12320) to inhibit protein synthesis. The immunoblotting was used to check TGFBR1 protein levels. The ratio of (TGFBR1/β-tubulin) was determined at different time points and expressed as percentages relative to the ratio at time 0. β-tubulin was used as a control protein.

### Co-immunoprecipitation (Co-IP)

NHLF cells were used for endogenous Co-IP experiments and cells at 48 h after infection by lentivirus containing overexpression constructors as indicated for exogenous Co-IP. Cells were lysed in triton lysis Buffer (TLB) by sonication on ice and the protein lysis was collected by centrifugation at 12000×*g*, 30 min, 4 °C. Following, 1 mg of protein lysis was mixed with 30 µl protein A/G and incubated 1.5 h on a rotator at 4 °C to eliminate nonspecific binding proteins. After centrifugation at 1000×*g*, the supernatant was incubated with 1 µg anti-flag antibody, anti-EZH2 (Santa Cruz, sc-13725s) and mouse IgG (negative control, (Beyotime, A7016) on the rotator at 4 °C overnight. Following 50 µl protein A/G was used to pull down antibody-conjugated proteins, then washing beads with TLB for more than three times. Finally, beads were resuspended in equal volume of 2 × loading and boiled at 95 °C for 10 min to separate the protein and beads. Samples were then analysed by immunoblotting.

### Chromatin immunoprecipitation (ChIP) assay

ChIP assay was conducted by using the ChIP assay kit (Beyotime, P2078) according to the manufacture-instruction. Briefly, cells grown to 80–90% conference in 100 mm dishes were washed in cold DPBS twice and cross-linked by 1% formaldehyde for 10 min at 37 °C. The cross-linking fixation was stopped by adding 1/10 volume of glycine solution (10×) and incubated for 5 min at room temperature. Cells were washed with pre-cold DPBS containing 1 mM PMSF three times and lysed in SDS lysis buffer on ice for 10 min followed by sonication. The supernatant was collected for immunoprecipitation with anti-H3K27me3 antibody (CST, 9733s) and IgG (Beyotime, A7028) control. The cross-linked DNA was purified with DNA Purification Kit (Beyotime, D0033) and examined by qPCR using specific primers which were listed in Additional file 2: Table S3.

### Cellular immunofluorescent staining (IF)

The NHLF cells were seeded and grown on glass coverslips coated with poly-l-lysine and fixed in 4% paraformaldehyde prior to being incubated with antibodies specific to Rcn3 (1:200, sigma, HPA050402) and α-actinin EZH2 (1:100, Santa Cruz, sc-13725s). The secondary anti-rabbit antibody conjugated to Alexa Fluor 488 (1:1000, ZSGB-BIO, ZF0511) and Alexa Fluor 594 (1:1000, ZSGB-BIO, ZF0513) was applied respectively to detect the immune signals, respectively. Fluorescence images were viewed with a Zeiss LSM980 confocal microscope.

### Statistical analysis

All data are presented as the mean ± SD. To compare continuous variables, the Kolmogorov–Smirnov test was used to test the normality of the data. Statistical comparisons between two groups were performed by the two-tailed Student’s *t*-test for normally distributed continuous variables or the Mann–Whitney U test for non-normally distributed continuous variables. Statistical comparisons of three or more groups were performed by using the one-way ANOVA (Bonferroni post hoc test) for normally distributed variables, one-way Kruskal–Wallis tests (Dunn’s post hoc test) for non-normally distributed variables, or two-way ANOVA (Tukey post hoc test). The statical significance was set at *p* < 0.05. All analyses were performed using GraphPad Prism 8.0 software.

## Results

### The upregulation of RCN3 in lung fibroblasts is critical for pulmonary fibrosis

**Fig. 1:**
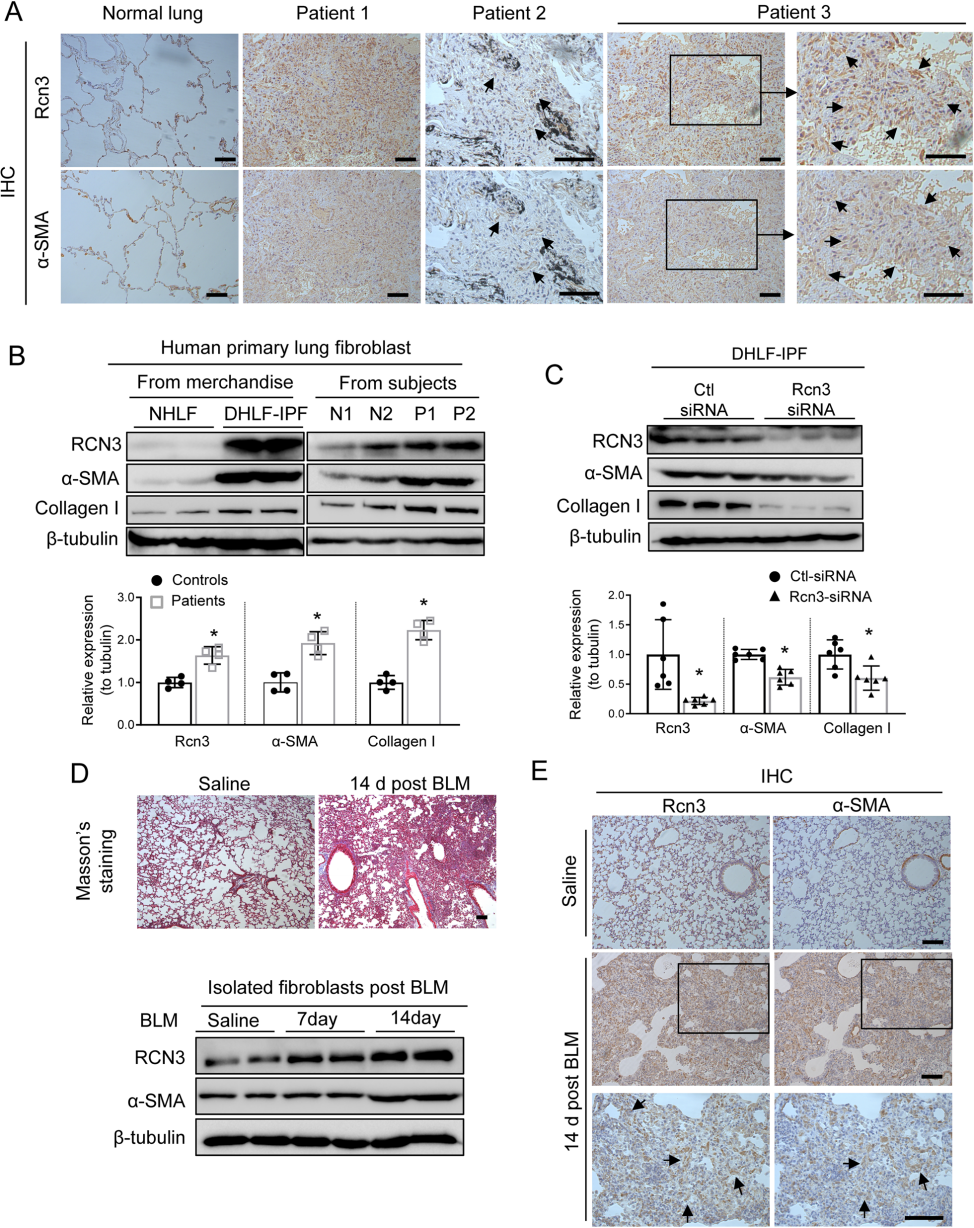
Rcn3 is markedly upregulated in lung fibroblasts during lung fibrosis and the depression of Rcn3 expression constricts the activation of lung fibroblasts. **A** IHC staining of α-SMA and Rcn3 on the continues sections from patient lung tissues with intestinal fibrosis and normal lung tissues indicates a dramatic increase of Rcn3 in α-SMA positive area (myofibroblasts). Arrows on higher magnification views show representative corresponding fields with both positive Rcn3 and α-SMA staining. **B** The immunoblot indicates marked upregulations of α-SMA, collagen I and Rcn3 in the fibrotic lung fibroblast from both merchandise and the patient subjects. β-Tubulin expression is the protein loading control and the ratios to tubulin expression are represented by the bar graphs relative to normal subjects (n = 4 per group, data presented as mean ± SD, *P < 0.05 versus normal subjects). **C** The immunoblotting shows that Rcn3 knockdown by small interfering RNA (siRNA) in fibrotic fibroblasts significantly constricts the expression of α-SMA and collagen I. The ratios to tubulin expression are presented in bar graphs as values relative to siRNA control (n = 6 independent biological replicates). **D** The Masson’s trichrome staining shows manifest lung fibrosis 14 days post the intratracheal instillation of bleomycin (up panel); the immunoblot indicates the markedly increased expressions of a-smooth muscle actin (α-SMA) and Rcn3 in the lung fibroblasts isolated from these mice (lower panel). **E** Immunohistochemistry (IHC) staining on the continues lung sections using anti-α-SMA and anti-Rcn3 antibodies show that the upregulated Rcn3 mainly localised in α-SMA positive area (myofibroblasts) after bleomycin treatment; arrows on higher magnification views show representative corresponding fields with both positive Rcn3 and α-SMA staining (lower panels). Data presented as mean ± SD, *P < 0.05 versus siRNA control). Two-tailed Student’s t-test for statistical comparisons between two groups. *BLM* bleomycin, *IHC* immunohistochemistry, *HNLF* normal human lung fibroblast, *DHLF-IPF* disease human lung fibroblasts-idiopathic pulmonary fibrosis, *Ctrl* control. Scale bars = 100 µm

First, we examined RCN3 expression in lung fibroblast (LF) from IPF patients. Immunohistochemical (IHC) on the continues sections from patient lungs with intestinal fibrosis showed prominent RCN3 induction in α-SMA positive area, whereas Rcn3 positive staining in normal lung tissues was primarily in the corner of alveoli (suggestive of AECIIs) in line with our previous publication [21] (Fig. 1A). Additionally, we explored the published single-cell RNA-seq on IPF patients (Sci. Adv. 6, eaba1983, 2020) and found a remarkable upregulation of Rcn3 in myofibroblasts rather than other cell types [26] (Additional file 1: Fig. S1A). Moreover, RCN3, along with α-SMA and collagen-I, was upregulated in disease lung fibroblasts derived from an IPF patient (DHLF-IPF, from merchandise Lonza) versus normal human lung fibroblast (NHLF, from merchandise Lonza), which was consistent with observations in fibroblasts isolated from patient lungs (Fig. 1B). Furthermore, RCN3 knockdown remarkably diminished the expressions of α-SMA and collagen-I in DHLF-IPF (Fig. 1C).

Next, we examined Rcn3 expression in mouse fibrotic lung fibroblasts. Intratracheal bleomycin instillation caused prominent pulmonary intestinal fibrosis (with isolated fibrotic foci) as indicated by Masson’s trichrome and IHC staining of α-SMA (Fig. 1D, E, Additional file 1: Fig. S1B). In the primer lung fibroblasts from these mice, Rcn3 was remarkably upregulated along with α-SMA (Fig. 1D). IHC staining on the continuous lung sections further confirmed that the striking increase of Rcn3 was localised in α-SMA positive area (Fig. 1E and Additional file 1: Fig. S1B). These findings on the patient and the mouse model suggested a critical role of Rcn3 upregulation in lung fibroblast during IPF.

### The selective repression of Rcn3 expression in fibroblast ameliorates bleomycin-induced lung fibrosis

**Fig. 2:**
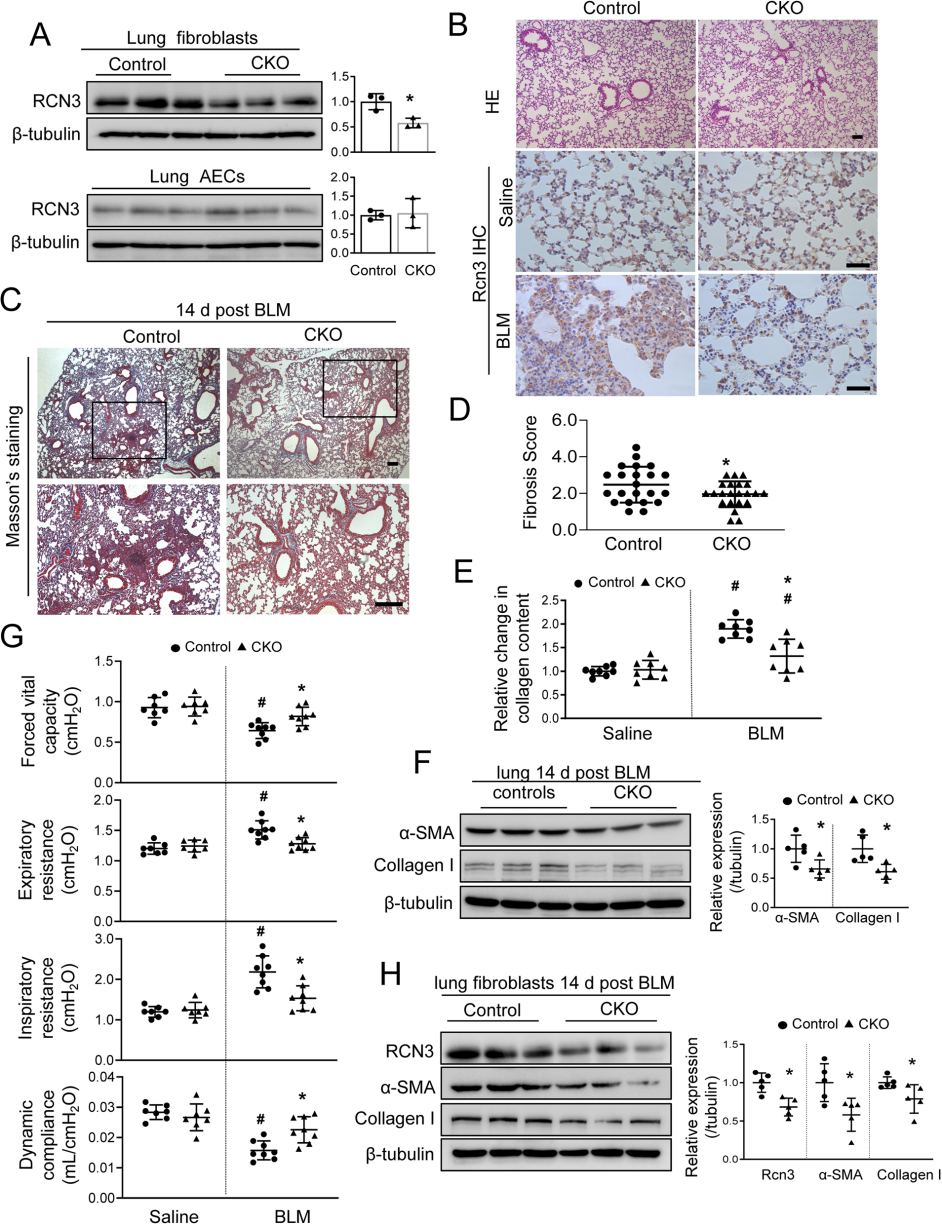
Mice with Rcn3 repression in fibroblast show ameliorated lung fibrosis induced by intratracheal bleomycin instillation. **A** Immunoblotting of Rcn3 in lung fibroblast and AECs isolated from the same CKO or control mice. **B** The HE staining of lung sections (up-panel); Immunohistochemistry of Rcn3 on sections from CKO and control lungs following the administrations of bleomycin or saline (lower-panel). **C** The Masson’s trichrome staining on lung sections at 14 days after bleomycin indicates repressed interstitial lung fibrosis in CKO versus control littermates. **D** The interstitial fibrosis was scored on the Masson’s trichrome-stained lung sections (n = 22 mice per group). **E** Lung collagen content from the right middle lobe was quantitated by total collage assay and presented as relative changes to the saline-treated control mice (n = 8 mice per group). **F** The immunoblot analyses of α-SMA and collagen I in the lung at 14 days post bleomycin instillation; the ratios to tubulin expression are presented in a dot graph as values relative to those of control littermates (n = 5 mice per group). **G** CKO mice exhibit alleviated bleomycin-induced alteration of pulmonary functional indexes, including forced vital capacity (FVC), Expiratory resistance (Re), inspiratory resistance (Ri) and dynamic compliance (Cdyn) (n = 7–8 mice per group). **H** The immunoblot analyses of Rcn3, α-SMA and collagen I in the lung fibroblast isolated from bleomycin-treated mice at 14 days post-instillation; the ratios to tubulin expression are presented in dot graph as values relative to those of control littermates (n = 5 mice, *P < 0.05 versus controls). Data are presented as the mean ± SD with statistical analysis performed by unpaired student’s t-tests or two-way ANOVA (Tukey post hoc test) as appropriate. *p < 0.05 versus control littermates in the same treatment group, ^#^p < 0.05 versus respective saline. *CKO* conditional knockout, *BLM* bleomycin. Scale bars = 100 µm

The role of Rcn3 in LF during lung fibrosis was further investigated by using the mouse model with fibroblast-selective *Rcn3* repression (CKO), which were generated by crossing the fibroblast-specific protein (FSP)1-Cre and Rcn3^flox/flox^ mice. The CKO mice (FSP1-Cre/Rcn3^f/f^) developed normally and their littermates (Rcn3^f/f^) served as controls (Additional file 1: Fig. S2). The immunoblotting assay showed that Rcn3 expression was remarkably repressed in LFs, but not in ACEs, isolated from the same CKO lung (Fig. 2A, Additional file 1: Fig. S2C). The ICH of Rcn3 further confirmed the declined Rcn3 staining inside the lung fibrotic foci from intratracheal bleomycin-treated CKO mice, whereases the Rcn3 staining in corner of alveoli (ACEs) and around the blood vessels (endothelial cells) was intact in these mice (Fig. 2B), which was consistent with a previous study that Fsp-1 is relatively fibroblast specific in the lung [27]. Since the previous study reports that Fsp1 may also express in immunocytes and hematopoietic cells [28], we checked the inflammatory condition in the lung to evaluate the potential nonspecific effects of FSP1-Cre mediated Rcn3 deletion. The CKO mice exhibit normal gross morphologies including body weight, motor activity and responses to painful stimuli, normal red blood cell (RBC) count, and comparable levels of IL-1β, TNFα and MCP1 mRNAs in the lung (Additional file 1: Fig. S2A, B).

In response to bleomycin instillation, CKO mice showed attenuated bleomycin-lung fibrosis versus controls, as indicated by constricted fibrotic foci formation and parenchymal distortion, which was in line with decreases in the semiquantitative fibrotic score, hydroxyproline content and the expressions of α-SAM, collagen I and Cyclin D1 (Fig. 2C–F, Additional file 1: Fig. S2D). Consistently, the bleomycin-induced decline of pulmonary function was significantly ameliorated in CKO mice, evidenced by limited changes of forced vital capacity (FVC), expiratory resistance (Re), inspiratory resistance (Ri) and dynamic compliance (Cdyn) (Fig. 2G). Additionally, although there have been reports of the non-specific effects of FSP1-Cre in immunocytes [28], CKO and control lungs showed comparable levels of IL-1β, TNFα and MCP1 at acute/fibrotic phases (3, 7 and 14 days) after bleomycin exposure, suggesting attenuated fibrosis in CKO lung was not due to decreased inflammation (Additional file 1: Fig. S3). Furthermore, the isolated LFs from CKO lungs exhibited significantly decreased Rcn3, α-SAM, collagen-I and Cyclin-D1 expressions, but not TGFβ1 (Fig. 2H, Additional file 1: Fig. S5). These observations suggested that Rcn3 induction in LFs was critical for bleomycin-induced fibrosis.

### RCN3 induction by TGFβ1 in an ER stress-dependent manner is essential for LF functional activation and Rcn3 upregulation blunted antifibrotic effects of pirfenidone and nintedanib

**Fig. 3:**
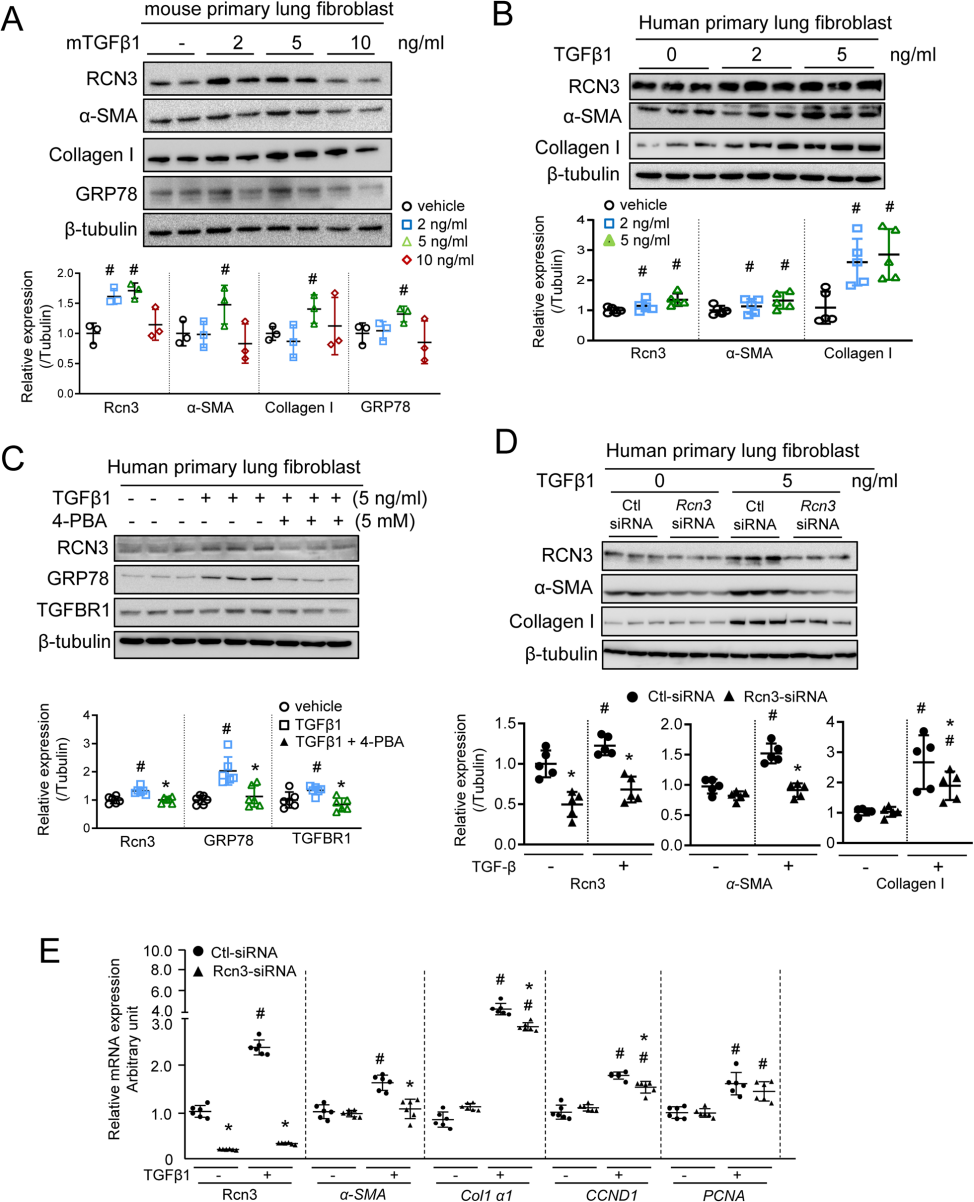
TGFβ1 exposure induced Rcn3 expression in ER Stress dependent manner and Rcn3 induction are critical for the activation of pulmonary fibroblast. The immunoblot analyses of the mouse (**A**) and human (**B**) lung fibroblast show the upregulations of Rcn3, α-SMA, collagen I, and GRP78 in response to TGFβ1 exposure at indicated concentrations. The ratios to tubulin expression are presented in dot graph relative to vehicle control (n = 3–5 independent biological replicates, ^#^P < 0.05 versus vehicle). **C** The ER-stress inhibitor 4-PBA significantly blunts the TGFβ1-induced upregulation of GRP78, Rcn3 and TGFBR1. The ratios to tubulin expression are presented in dot graph relative to vehicle control (n = 5 independent biological replicates, ^#^P < 0.05 versus vehicle). **D** The immunoblot analyses indicate that Rcn3 depression by siRNA markedly constricts TGFβ1-induced upregulations of α-SMA and collagen I. The ratios to tubulin expression are presented in dot graph relative to the vehicle-treated Ctl-siRNA group (n = 5 independent biological replicates). **E** qPCR analyses of Rcn3, Col1a1, αSMA, CCND1 and PCNA in human lung fibroblast exposed to TGFβ1 (5 ng/ml) for 24 h. The data were normalised to the GAPDH content and analysed by the 2^−∆∆Ct^ method relative to the vehicle Ctl-siRNA group (n = 6 independent biological replicates per group). Data presented as mean ± SD; 2-way ANOVA (Tukey post hoc test) was performed. ^#^p < 0.05 vs vehicle treatment at same siRNA group; **D** and **E** *p < 0.05 vs Ctl-siRNA at same treatment. *CKO* conditional knockout, *BLM* bleomycin, *Ctl-siRNA* control-siRNA, *Col1a1*: collagen I a1, *CCND1* Cyclin D1, *PCNA* proliferating cell nuclear antigen

**Fig. 4:**
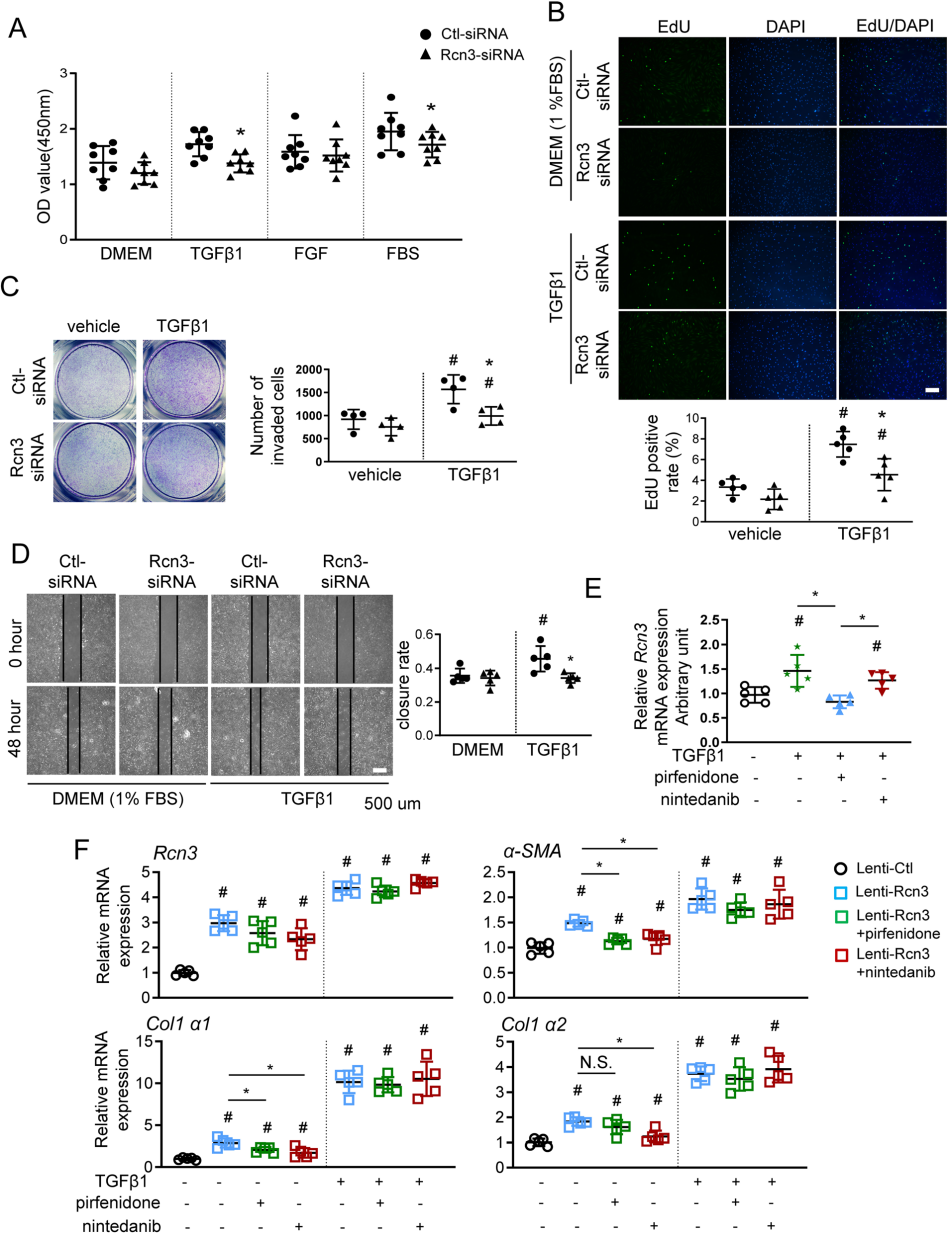
Rcn3 is essential for TGFβ1-induced HLF proliferation and migration and activation as well as associated with the resistance of antifibrotic effects of pirfenidone and nintedanib. **A** Cell Counting Kit-8 (CCK8) assay was used to analyse the proliferation of NHLFs with Ctl-/Rcn3-siRNA in response to TGFβ1 (5 ng/ml), FGF (50 ng/ml) or FBS serum (10%) for 24 h (n = 8 independent biological replicates). **B** Proliferation of NHLFs with *Rcn3-* and Ctl-siRNA upon TGFβ1 exposure was examined by EdU incorporation assay. The representative views with EdU positive (green spots) were shown in the up-panel and the percentage of EdU positive was presented as dot graph (n = 5 independent biological replicates). **C** The transwell migration assay examined TGFβ1-induced cell migration in NHLF cells with Rcn3- and Ctl-siRNA; the representative whole-cell pictures were shown in the up-panel. The number of invaded cells was counted and presented in a dot graph (n = 4 independent biological replicates). **D** Scratch assay was used to analyse cell migration in response to TGFβ1 exposure for 48 h; the representative views were shown in the up-panel. The closure rates were calculated and presented in a dot graph (n = 5 independent biological replicates). **E** qPCR analyses of *Rcn3* in NHLF co-treated with TGFβ1, pirfenidone and nintedanib as indicated for 24 h: TGFβ1 at 5 ng/ml, pirfenidone at 100 ng/ml, nintedanib at 150 ng/ml. **F** qPCR analyses of *αSMA*, *Col1a1*, and *Col1a2* in NHLF with Rcn3 overexpression in response to TGFβ1, pirfenidone and nintedanib for 24 h as indicated. Data are presented as the mean ± SD with statistical analysis performed by unpaired student’s *t-*tests*,* one-way ANOVA (Tukey post hoc test) and two-way ANOVA (Tukey post hoc test) as appropriate. ^#^p < 0.05 vs vehicle treatment at same siRNA group; *p < 0.05 vs Ctl-siRNA at same treatment. In **E** and **F**, ^#^p < 0.05 vs Lenti-Ctl vehicle controls, *p < 0.05 as indicated. *NHLF* normal human lung fibroblast, *CM* culture medium, *Ctl-siRNA* control-siRNA, *FGF* fibroblast growth factors, *FBS* foetal bovine Serum (FBS)

To investigate whether RCN3 induction during lung fibrosis was associated with TGFβ1 signalling, NHLF and mouse lung fibroblast (MLF) were treated with TGFβ1 at different concentrations. TGFβ1-exposure at 2 and 5 ng/ml doses enhanced the expressions of RCN3, α-SAM, collagen-I and ER stress marker GRP78 (Fig. 3A, B). Interestingly, ER stress inhibitor 4-phenylbutyrate (4-PBA) strikingly attenuated the inductions of GRP78 and RCN3 in response to TGFβ1-exposure, suggesting an ER stress-dependent RCN3 induction (Fig. 3C and Additional file 1: Fig. S4A). Furthermore, RCN3 knockdown by siRNA suppressed the activation of fibroblast induced by TGFβ1, but not FGF, as demonstrated by diminished expressions of α-SAM, collagen-I and Cyclin-D1 (Fig. 3D, E, Additional file 1: Fig. S4B). CCK8 and EdU incorporation assays consistently showed that RCN3 deficiency significantly blunted NHLF proliferation in response to TGFβ1 and FBS (10%) rather than FGF (Fig. 4A, B). RCN3 deficiency also caused a notable depression of TGFβ1-induced migration, as indicated by transwell and scratch assays (Fig. 4C, D). These results indicated the importance of Rcn3 upregulation for activating LF upon TGFβ1-exposure.

Next, we investigated whether RCN3 would exhibit the difference in the anti-profibrotic effects of Nintedanib and Pirfenidone, because Nintedanib inhibits multiple receptor tyrosine kinases (FGFR and VEGFR) and Pirfenidone primarily inhibits the TGFβ1 signalling [29]. TGFβ1-induced upregulations of *RCN3* were blunted by pirfenidone rather than nintedanib, although both of two drugs significantly inhibited TGFβ1-induced upregulations of *α-SMA*, *Col1a1* and *Col1a2* (Fig. 4E and Additional file 1: Fig. S5)*.* Interestingly, Rcn3 overexpression-caused NHLF activation was significantly bunted by both drugs. Furthermore, it is worth nothing that RCN3 overexpression significantly dampened the anti-fibrotic effects of both drugs against TGFβ1-induced LF activation, suggesting that Rcn3 upregulation could restrain their therapeutic effects for IPF (Fig. 4F).

### RCN3 was involved in the activations of both canonical and non-canonical TGFβ1 signalling in lung fibroblasts

**Fig. 5:**
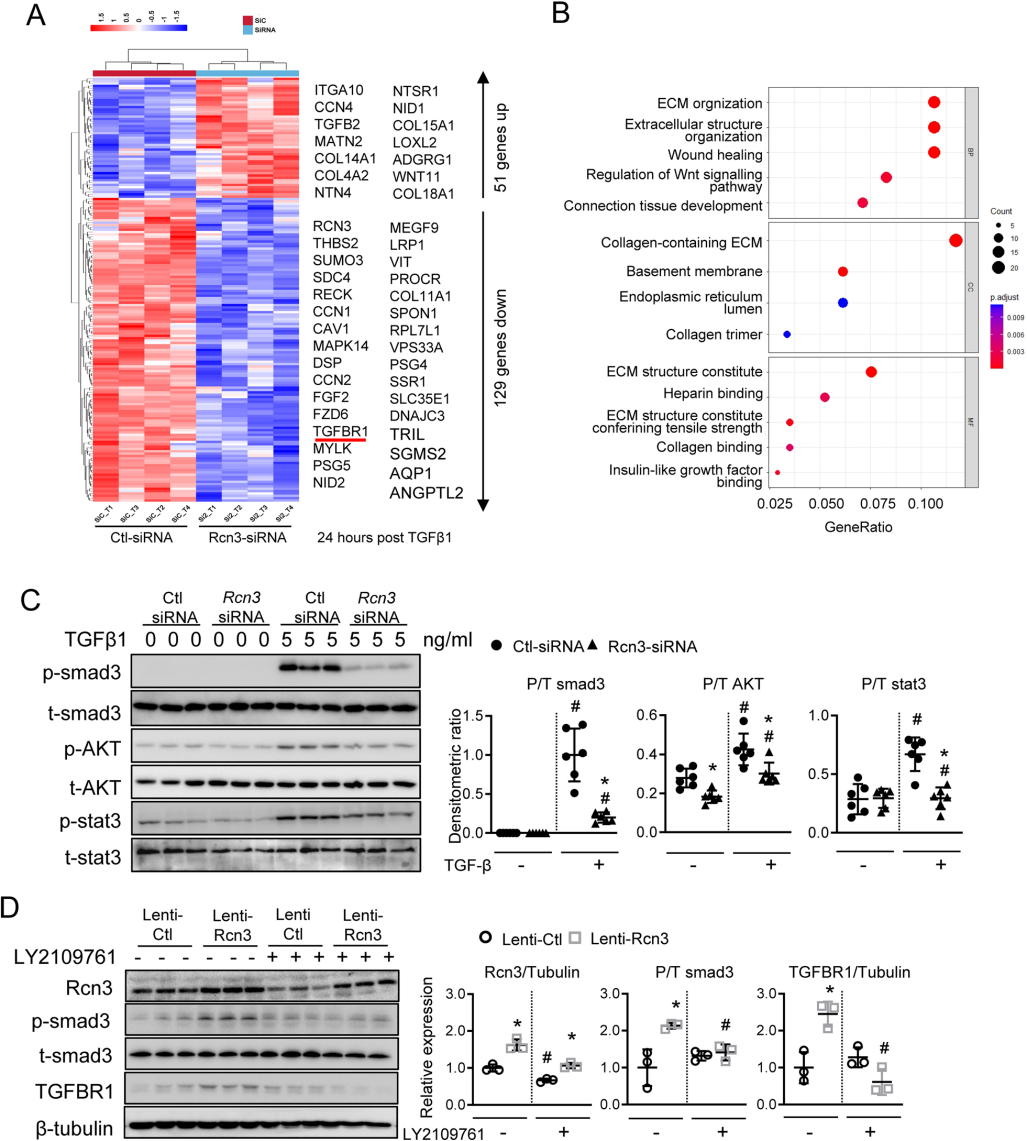
Rcn3 deficiency repressed the activations of both canonical and non-canonical TGFβ1 signalling in lung fibroblasts. **A** The RNA-Sequencing analyses of the total RNA from NHLF cells with *Rcn3*-siRNA (Si-T) and Ctl-siRNA (SiC-T) after TGFβ1 exposure for 24 h (n = 4 per group). A total of 180 differentially-expressed genes (DEGs) with the foldchange (FC) > 1.2 and FDR < 0.05 were identified between Si-T versus SiC-T. Hierarchical clustering presented the gene expression profiles separated based on *Rcn3* knockdown. **B** The Gene Ontology analysis on these DEGs prioritised by strength (Gene-ratio: the ratio of the number of deferentially expressed genes between the number of genes associated the GO term) and FDR p values corrected by BH procedure (p < 0.05); The most affected processes were presented. *BP* biological process, *CC* cellular component, *MF* molecular function. **C** Immunoblot of p-smad3, t-smad3, p-AKT, t-AKT, p-stat3 and t-stat3 in the NHLF cells with *Rcn3-* and Ctl-siRNA in response to TGFβ1 exposure. The densitometric ratios of p/t-smad3, p/t-AKT and p/t-stat3 were calculated and presented as dot graphs (n = 6 independent biological replicates). **D** Immunoblot of Rcn3, p-smad3, t-samd3 and TGFBR1 in the NHLF cells infected with lentivirus-Rcn3 (Rcn3 overexpression) and lentivirus-control in response to the TGFβ receptor inhibitor, LY2109761. The densitometric ratios of Rcn3/tubulin, P/T smad3 and TGFBR1/tubulin are plotted in dot graphs as values relative to untreated lentivirus-control (n = 3 independent biological replicates). Data are presented as the mean ± SD with statistical analysis performed by two-way ANOVA (Tukey post hoc test). ^#^p < 0.05 vs vehicle treatment at same siRNA or lentivirus group; *p < 0.05 vs Ctl-siRNA or lenti-Ctl at same treatment. *NHLF* normal human lung fibroblast, *Ctl-siRNA* control-siRNA, *lenti-Ctl* lentivirus-control, *lenti-Rcn3* lentivirus-Rcn3, *p-smad3* phospho-Smad3, *t-smad3* total-smad3, *P-AKT* phospho-AKT, *t-AKT* total AKT, *p-stat3* phospho-stat3, *t-stat3* total-stat3, *P/T* phospho-/total-protein

To further dissect the regulatory mechanism of RCN3 in TGFβ1 signalling, TGFβ1-treated NHLF bearing RCN3-siRNA (si-T) and control-siRNA (siC-T) were subjected to RNA-Sequencing analysis. A total of 181 differentially-expressed genes (51 upregulation, 129 downregulation genes) were identified (foldchange (FC) > 1.2, FDR < 0.05), including genes involved in fibrosis and cell cycle, suggesting that Rcn3 deficiency could affect both canonical and non-canonical TGFβ1 signalling (Fig. 5A). Most RCN3 deficiency-affected processes enriched by Gene Ontology (GO) analysis were primarily associated with fibrosis including ECM organisation, wound healing and ECM structure constitutes (Fig. 5B). Of note, *TGFBR1* was found in the down-regulation panel, suggesting a potential role of RCN3 in regulating the initiation of TGFβ1 signalling. This hypothesis was further supported by the observation that RCN3 knockdown markedly blunted both canonical and non-canonical TGFβ1 downstream signalling pathways (Fig. 5C). Moreover, the TGFBRs inhibitor, LY2109761, completely suppressed the activation of smad3 caused by RCN3 overexpression (Fig. 5D).

### RCN3 facilitates TGFβ1 signalling by the transcriptional increase of TGFBR1, establishing a positive feedback loop

**Fig. 6:**
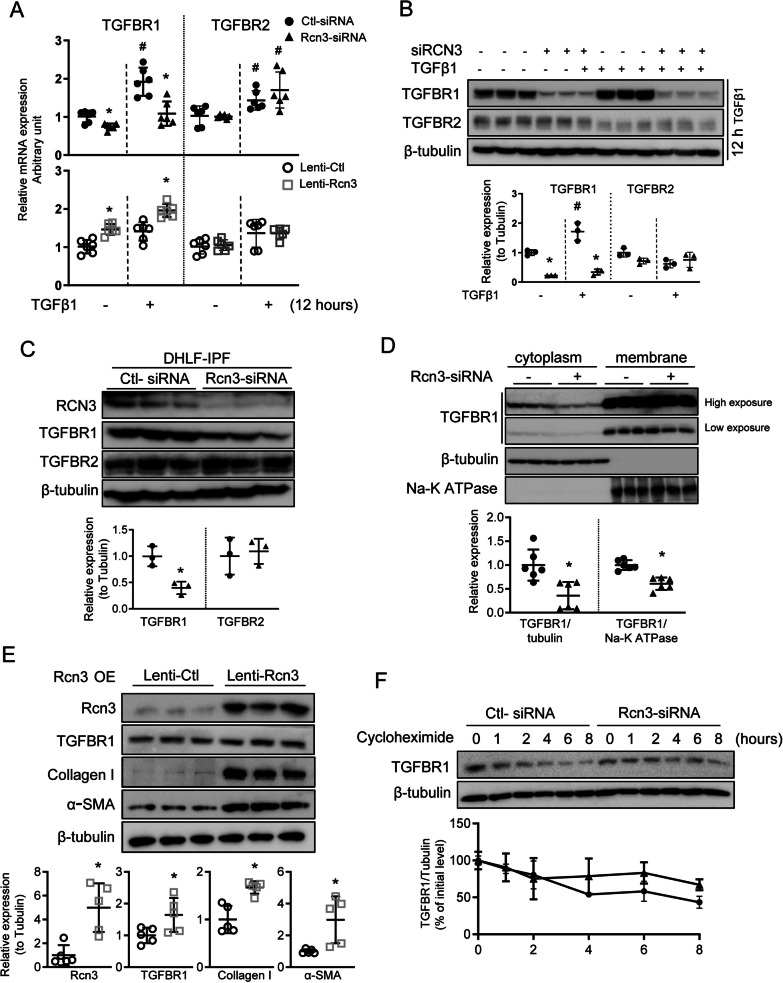
Rcn3 facilitates TGFβ1 signalling by transcriptionally maintaining the expression of TGFBR1. **A** qPCR analyses of *TGFBR1* and *TGFBR2* in NHLF with Rcn3 knockdown or overexpression upon TGFβ1 exposure for 12 h. The qPCR data were normalised to the *GAPDH* content and analysed by the 2^−∆∆Ct^ method relative to the vehicle Ctl-siRNA group (n = 6 independent biological replicates). The immunoblot assay indicates that Rcn3 knockdown by siRNA significantly inhibits the expression of TGFBR1 with intact TGFBR2 expression in NHLF upon TGFβ1 exposure for 12 h (**B**) and in DHLF-IPF (**C**). The ratios to tubulin expression are presented in dot graph relative to Ctl-siRNA (n = 3 independent biological replicates). **D** The immunoblot of cytoplasmic and membrane protein fractions from NHLF with *Rcn3*-siRNA and Ctl-siRNA. The β-tubulin expression and Na–K ATPase are as loading controls for cytoplasmic and membranous proteins, respectively. Cytoplasmatic-TGFBR1/tubulin and membrane-TGFBR1/Na–K ATPase ratios are presented in dot graph relative to Ctl-siRNA (n = 6 independent biological replicates). **E** The overexpression of Rcn3 causes marked increases in TGFBR1, collagen I, and α-SMA in NHLF. The ratios to tubulin expression are presented in dot graph relative to lenti-Ctl (n = 5 independent biological replicates). **F** Knockdown of Rcn3 failed to change TGFBR1 protein degradation in NHLF, as indicated by cycloheximide chase assay. (n = 3 independent experiments). Data are presented as the mean ± SD, and differences between two groups were analysed by unpaired student’s t-tests or Mann–Whitney U-tests as appropriate. *P < 0.05 versus Ctl-siRNA or lenti-Ctl. *NHLF* normal human lung fibroblast, *DHLF-IPF* disease human lung fibroblasts-idiopathic pulmonary fibrosis, *Ctrl-siRNA* control-siRNA, *lenti-Rcn3* lentivirus-Rcn3, *lenti-Ctl* lentivirus-control

We further investigated the regulatory mechanism of RCN3 in moderating TGFβ1-TGFBR1 signalling. We found that primary lung fibroblasts from CKO mice had significantly reduced expression of TGFBR1, but not TGFβ1, along with diminished Rcn3 expression (Additional file 1: Fig. S2C). The significantly altered mRNA level of *TGFBR1,* but not *TGFBR2,* was also observed in NHLF with either RCN3 knockdown or overexpression (Fig. 6A). Consistently, RCN3 knockdown significantly diminished the protein level of TGFBR1 in both NHLF and DHLF-IPF. NHLF bearing RCN3 knockdown also were unable to maintain the level of TGFBR1 upon TGFβ1 stimulation (Fig. 6B, C, Additional file 1: Fig. S6). The reduction of TGFBR1 protein was observed in both cytoplasm and membrane fractions (Fig. 6D), which was in line with blunted TGFβ1 signalling. Additionally, RCN3 overexpression significantly upregulated TGFBR1 expression, accompanied with the inductions of fibrotic markers (Fig. 6E). Furthermore, protein stability by cycloheximide chase assay showed an unchanged protein degradation rate of TGFBR1 in RCN3 deficiency NHLF (Fig. 6F), suggesting a transcriptional regulation of TGFBR1 by RCN3. Additionally, RCN3 deficiency failed to change *TGFBR2* transcription, validated by using three sets of qPCR primers targeting different regions in either Rcn3-siRNA or shRNA-targeted NHLFs (Additional file 1: Fig. S6).

Taken together, the TGFβ1-RCN3-TGFBR1 positive-feedback loop was proposed based on the evidences: TGFβ1-exposure upregulated RCN3 and TGFBR1; RCN3 deficiency diminished TGFβ1-induced TGFBR1 upregulation; RCN3 overexpression upregulated TGFBR1 leading to TGFβ1 signalling activation, which was repressed by TGFBR inhibitor LY2109761.

### The TGFβ1 exposure promoted RCN3-EZH2 interaction which restrained nuclear EZH2 level and decreased EZH2/H3K27me3 enrichment at the TGFBR1 promoter region

**Fig. 7:**
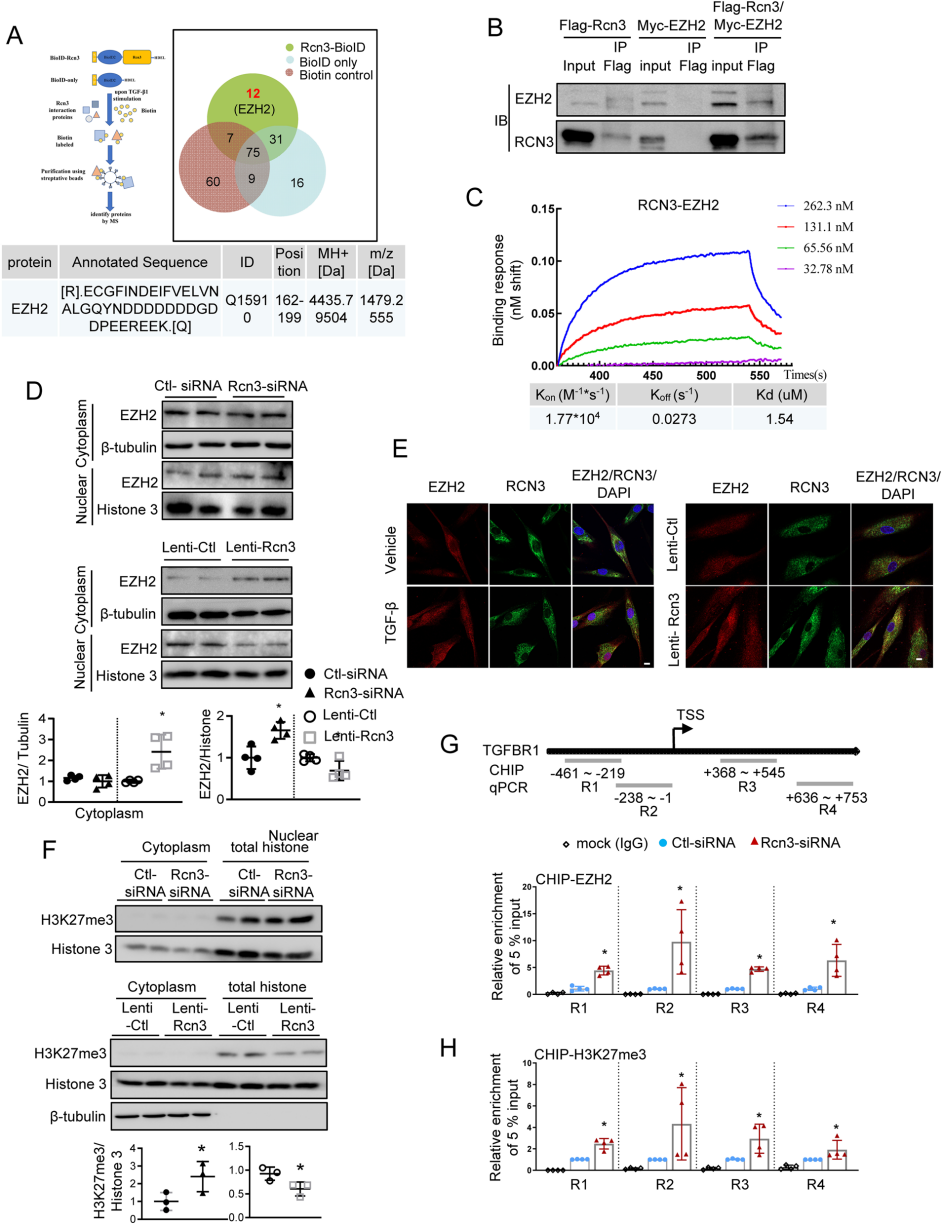
TGFβ1 exposure promoted Rcn3-EZH2 interaction which restrained nuclear EZH2 level and decreased EZH2/H3K27me3 enrichment at the TGFBR1 promoter region. **A** A schematic diagram of BioID labelling strategy (up-left panel). The BioID-Rcn3 fusion protein was constructed by inserting *Rcn3* (without signal peptide) into the C-terminal of BioID2 and the signal peptide fragment of Rcn3 into of N-terminal of BioID2; BioID-Rcn3 and BioID-only (bearing Rcn3 signal peptide in the N-terminal) vectors were transfected into NHLF. Upon TGFβ1 exposure, selective biotinylation of proximal proteins is followed by stringent cell lysis for streptavidin-affinity purification and identification by LC–MS/MS. By eliminating the common proteins in BioID-Rcn3, BioID-only and Biotin controls, 12 proteins were identified as specific potential interaction proteins of Rcn3, including EZH2 (right panel). The amino acid sequence of the EZH2 peptide fragment identified by MS was shown in a table (lower panel). **B** Co-IP validated Rcn3-EZH2 interaction in NHLF co-transfected with Flag-Rcn3, Myc-EZH2 or Flag-Rcn3 + Myc-EZH2. **C** Biolayer interferometry (BLI) studies by using recombination human Rcn3 and EZH2 protein indicated the direct binding between Rcn3 and EZH2 and the binding with a clear concentration gradient with an affinity (KD) of 1.54 µM. BLI binding representative of at least 3 independent traces. **D** The immunoblot of EZH2 in nuclear protein fraction from NHLF. The ratios to histone 3 expression were presented as dot graph relative to controls (n = 4, independent biological replicates). **E** Immunofluorescence staining of Rcn3 (green) and EZH2 (red) on the NHLF cells in response to TGFβ1 exposure and NHLF with Rcn3 overexpression. The DAPI (blue) is used for nuclear staining. TGFβ1 treatment and Rcn3 overexpression promote Rcn3-EZH2 interaction in the cytoplasm (the yellow dots in the merge views). Scale bar: 10 μm. **F** The immunoblot of H3K27me3. The H3K27me3/Histone 3 ratio was present as a dot graph relative to Ctl-siRNA or Lenti-Ctl (n = 3, independent biological replicates). **G**, **H** chromatin immunoprecipitation (ChIP) assay was used to analyse H3K27me3 in TGFBR1 promoter in NHLF with Rcn3-siRNA. Schematics of TGFBR1 and related four sets of ChIP qRT-PCR primers (up panel). qPCR analysis of TGFBR1 promoter regions conducted with 4 sets of specific primers (R1-R4) after a ChIP assay using anti-H3K27me3 antibody or the IgG isotype control. The results were presented as fold enrichments relative to Ctl-siRNA (n = 4 independent biological replicates). **H** Schematic summary of the proposed profibrotic mechanisms, a positive-feedback loop TGFβ1-Rcn3-TGFBR1, by which Rcn3 contributes to the pathogenesis of pulmonary fibrosis. Data are presented as the mean ± SD with unpaired student’s *t-tests* or Mann–Whitney *U-tests* to compare two groups. *P < 0.05 versus Ctl-siRNA or lenti-Ctl. *Co-IP* co-immunoprecipitation, *NHLF* normal human lung fibroblast, *Ctrl-siRNA* control-siRNA, *lenti-Rcn3* lentivirus-Rcn3, *lenti-Ctl* lentivirus-control

To further determine the regulatory mechanism of Rcn3 in TGFBR1 transcription, the BioID labelling was used to screen physiological interaction proteins of RCN3 in living NHLF cells in response to TGFβ1-exposure. After eliminating the common proteins in BioID-RCN3, BioID-only and Biotin controls, a total of 12 proteins were identified as specific interaction proteins of Rcn3 upon TGFβ1 stimulation (Fig. 7A). One of these proteins is Enhancer of zeste homolog 2 (EZH2) which is an epigenetic methyltransferase in polycomb repressive complex 2 (PRC2) and has been shown to suppress TGFBR1 transcription by catalysing tri-methylation of histone H3 at Lys 27 (H3K27me3) at the promoter region [16]. The RCN3-EZH2 interaction was confirmed by co-immunoprecipitation (Co-IP) by using exogenously expressed Flag-RCN3 and Myc-EZH2 (Fig. 7B). The quantitative assessment of the binding affinity by the Bio-Layer Interferometry (BLI, Octet-RED system) further confirmed their direct interaction as indicated by a concentration-dependent interaction of Rcn3 with EZH2 with an estimated dissociation constant (Kd) of 1.54 µM (Fig. 7C). Furthermore, we gained insight into RCN3-EZH2 complex formation from protein–protein docking analysis (Cluspro 2.0), exhibiting several high-confidence structural models of RCN3-EZH2 complex as rigid bodies with acceptable interaction weighted scores. The top potential models suggested that a segment of RCN3 (121–191 residues) seemed located in a pock-like structure of EZH2 (Additional file 1: Fig. S7).

EZH2 functions as a transcriptional suppressor through H3K27me3 within the nucleus, while immunofluorescence assay on Hela cells showed some co-localization of EZH2 and RCN3 within the cytoplasm (Additional file 1: Fig. S8). Furthermore, RCN3 knockdown significantly increased the level of nuclear EZH2 in HLF, while RCN3 overexpression decreased the level of nuclear EZH2 (Fig. 7D). Immunofluorescence on HLF showed more cytoplasmatic EZH2 than that in Hela cells; either TGFβ1 exposure or Rcn3 overexpression promoted the colocalization of Rcn3-EZH2 in the cytoplasm (Fig. 7E). In line with the change of nuclear-EZH2 level, RCN3 knockdown and overexpression markedly enhanced and diminished H3K27me3 level, respectively (Fig. 7F). Furthermore, the ChIP assay consistently indicated that RCN3 deficiency caused dramatically increased EZH2/H3K27me3 enrichment at the TGFBR1 promoter region (Fig. 7G, H). Therefore, RCN3-EZH2 interaction in cytoplasm likely plays a vital role in releasing EZH2/H3K27me3 epigenetic repression of TGFBR1 to maintain the TGFBR1 level during the receptor turnover upon TGFβ1 stimulation. The TGFβ1-RCN3/EZH2/H3K27me3-TGFBR1 is a kind of dynamic turnover modification for TGFβ1 signalling.

## Discussion

**Fig. 8:**
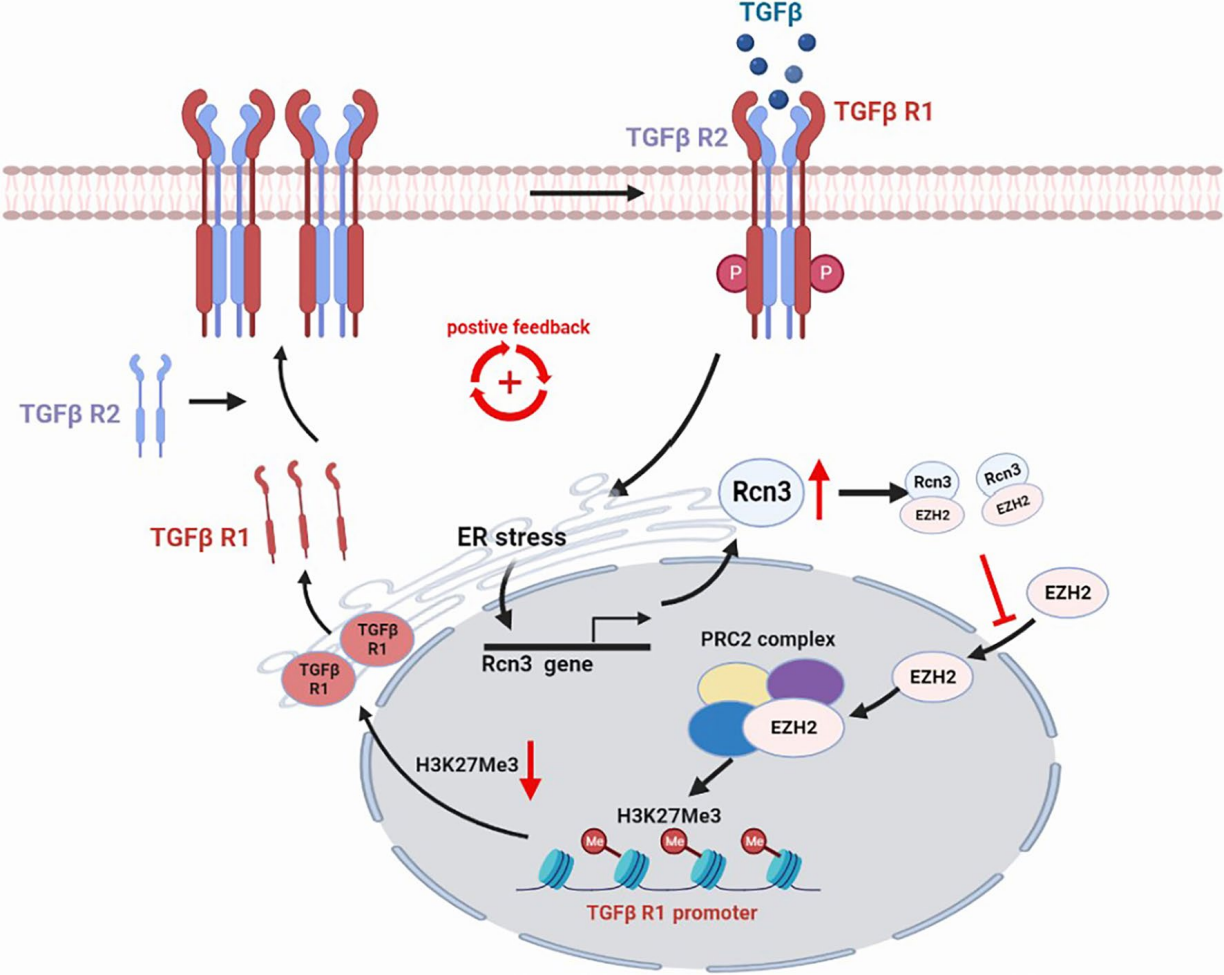
Schematic summary of the proposed profibrotic mechanisms, a positive feedback of TGFβ1 signalling, by which Rcn3 contributes to the pathogenesis of pulmonary fibrosis. Upon the activation of pulmonary injury-repair, TGFβ1 enhances Rcn3 expression in interstitial lung fibroblast through an ER stress signalling dependent manner; those increased Rcn3, in turn, detains EZH2 in the cytoplasm, leading to the decrease of H3K27me3 in TGFBR1 promoter region and the following enhancements of *TGFBR1* expression and TGFβ1 signalling

IPF is the most aggressive fibrotic ILD and lacks effective therapy due to unclear pathogenesis, so the discovery of novel regulators could shed light on developing alternative therapeutic strategies [30, 31]. Herein, we discovered a critical role of RCN3 in orchestrating fibroblast function during pulmonary fibrosis, as evidenced by key findings: (1) RCN3 was notably upregulated in fibroblasts from both patient and mouse fibrotic lungs, whereas RCN3 knockdown diminished activation of lung fibroblast from IPF patients. (2) The repression of Rcn3 in fibroblast ameliorated bleomycin-induced pulmonary fibrosis and dysfunction with suppressed LF activation. (3) the in vitro TGFβ1-exposure significantly upregulated RCN3 in an ER stress-dependent manner, while RCN3 knockdown diminished TGFβ1-induced fibrotic activation. Consistently, RCN3 overexpression promoted fibroblast activation and suppressed the effects of both pirfenidone and nintedanib against TGFβ1-induced fibroblast activation. (4) RCN3 facilitated fibroblasts activation by enhancing TGFβ1 signalling through transcriptional upregulation of *TGFBR1*, which is associated with reduced EZH2/H3K27me3 enrichment at *TGFBR1* promoter. (5) The physiological RCN3-EZH2 interaction released EZH2/H3K27me3 epigenetic repression of TGFBR1 upon TGFβ1 stimulation, suggesting a dynamic turnover modification. Taken together, these results depict a positive-feedback loop TGFβ1-RCN3-TGFBR1 as a critical mechanism in the pathogenesis of pulmonary fibrosis (shown as a schematic illustration in Fig. 8): upon injury-repair activation, TGFβ1 stimulation enhances RCN3 expression in interstitial lung fibroblast and such induction of RCN3 releases the EZH2/H3K27me3-dependent repression of TGFBR1 via RCN3-EZH2 interaction, leading to enhanced TGFBR1 expression and then persistent activation of TGFβ1 signalling.

### The RCN3-mediated positive feedback loop of TGFβ1 in lung fibroblast is likely a determinant for the imbalance of the pulmonary injury-repair process

The predominant cause of IPF is repeated alveolar epithelium injuries accompanied by the release of proinflammatory and profibrotic mediators, leading to an aberrant injury-repair process represented as progressive and irreversible interstitial fibrosis [2]. Our previous study using Rcn3 AECII-selective deletion mice indicates that Rcn3 in AECII is antifibrotic against bleomycin-induced pulmonary fibrosis via alleviating ER stress-induced AEC apoptosis [21]. Herein, observations on mice with fibroblast-selective Rcn3 repression indicate a profibrotic function of Rcn3 in lung fibroblast. Since the injury-repair process includes alveolar epithelial damage, repair and interstitial remodelling, Rcn3 likely facilitates the whole process by functioning in different roles in epithelium and fibroblasts. However, the aberrant fibrotic remodelling in IPF is largely due to the excessive fibroblast activation, so Rcn3 upregulation in fibroblast could be an essential trigger of shifting to imbalanced interstitial remodelling. This hypothesis was confirmed by the evidences: a striking induction of RCN3 was observed in fibroblasts from both patient and mouse fibrotic lungs; RCN3 overexpression promoted LF activation; Rcn3 deficiency markedly blunted DHLF-IPF activation and bleomycin-induced fibrosis.

The abundant evidence from human and animal studies demonstrates a central role of TGFβ1 in IPF pathogenesis [32]. Upon alveolar epithelial injury, TGFβ1 is the predominant profibrotic factor to orchestrate fibroblast functions, including differentiation, proliferation and ECM synthesis through smad and non-smad signalling pathways [33]. Herein, TGFβ1 exposure strikingly upregulates RCN3 in both human and mouse fibroblasts, while the suppression of RCN3 induction dampened both smad and non-smad signalling with depressed fibroblast activation. Interestingly, the RCN3 overexpression enhanced TGFβ1 signalling even without extra TGFβ1 exposure, which is associated with increased TRFBR1 expression rather than TGFβ1. Consistently, RCN3 deficiency transcriptionally diminished TGFBR1 expression upon TGFβ1 stimulation, suggesting that RCN3 is indispensable for maintaining persistent activation of TGFβ1 signalling via preserving membrane TGFBR1 level. These findings, for the first time, uncover a determinate role of the positive-feedback loop of TGFβ1-RCN3-TGFBR1 in lung fibrosis, as well as introduce a novel regulating mechanism of TGFβ1 signalling.

Since IPF is associated with a variety of pathways, including TGFβ1 and other tyrosine kinases pathways (PDGF, FGF and VEGF), The treatments for IPF require a multiple-targeting strategy, in which the TGFβ1 signalling is an essential pharmacological target. However, TGFβ1 acts in different functions in a variety of cell types, including epithelial, mesenchymal and inflammatory cells, so the direct inhibition of TGFβ1/TGFBR may be of inconsistent efficacy as well as cause side-effects such as increased inflammation [34]. The novel positive feedback TGFβ1-Rcn3-TGFBR1 loop plays a crucial role in the persistent activation of TGFβ1 signalling in fibroblasts. Therefore, targeting RCN3 could be an effective approach for treating IPF.

### The regulation of TGFBR1 rather than TGFBR2 is involved in the Rcn3-mediated positive feedback loop

A dynamic regulation of membrane TGFBR1/2 orchestrates TGFβ1 signalling through ligand-independent and ligand-dependent recycling [35, 36]. The dynamic regulation of TGFBR1 (the executor of TGFβ1 action) is essential to modulate the amplitude and duration of the response, and an imbalanced regulation can cause excessive signalling activation. Our results indicate an Rcn3-dependent transcriptional upregulation of TGFBR1 upon TGFβ1-exposure without altering the turnover and recycling. Therefore, during the injury-repair process, Rcn3 deregulation may cause persistent TGFBR1 expression, triggering balance toward hyperactivation of TGFβ1 signalling. Consistently, recent studies have highlighted the importance of increased TGFBR1 in fibroblast activation in IPF [12, 37]. Our results also showed that Rcn3 knockdown and overexpression altered TGFBR1 expression but not TGFBR2. Therefore, the Rcn3-mediated feedback loop is not involved with TGFBR2, which is consistent with previous studies that the expression and basolateral membranes trafficking of TGFBR1 and TGFBR2 are independently regulated [12, 35, 36].

In this study, we identified the Rcn3-EZH2 interaction in repose to TGFβ1 stimulation and uncovered a novel EZH2/H3K27me3-mediated epigenetic regulation of *TGFBR1* by Rcn3-EZH2 interaction. Consistently, a previous study showed that DNMT3A suppressed TGFBR1 transcription by recruiting EZH2 to *TGFBR1* promoter [16]. However, cancer studies showed that EZH2 regulated TGFBR2 via the H3K27me3-dependent mechanism, by which hypoxia and YAP/TAZ signal attenuated TGFBR2 expression in prostate and lung cancer, respectively [14, 38]. Inconsistently, we did not find that Rcn3-EZH2 could alter TGFBR2 transcription, which might be due to distinct regulatory mechanisms in cancer cells and fibroblasts.

Additionally, increasing evidence shows that EZH2 can function as both H3K27me3-dependent transcriptional suppressor and H3K27me3-independent co-activator [39–43]. Several regulatory mechanisms have been identified in different cellular contexts. For example, the phosphorylation of EZH2 at K307 by TAK1 in injured respiratory epithelium or at T487 by CDK in cancer triggered the switch from H3K27me3-dependent to -independent function [41, 43]. Conversely, the methylation of EZH2 at K307 enhanced the H3K27me3-dependent repression of tumour-suppressor genes, promoting breast cancer cell proliferation and invasion [40]. These various regulatory mechanisms occurring in distinct cellular contexts could explain why the Rcn3-EZH2 axis failed to regulate TGFBR2 transcription, as well as suggest the specificity of the Rcn3-mediated loop in regulating fibroblast function.

### The enhanced RCN3 expression in IPF patients could lead to the resistance to pirfenidone and nintedanib treatments

Pirfenidone and nintedanib are recently approved anti-profibrotic drugs for IPF and fibrotic ILD treatments [5, 6, 29, 44]. The anti-fibrotic effect of pirfenidone is mainly attributed to the inhibitions of both TGFβ1 production and signalling activation, while nintedanib primarily functions as the receptor tyrosine kinases inhibitor for profibrotic mediators, FGFR, PDGF and VEGFR [6, 29]. In line with different phonological mechanisms, pirfenidone, rather than nintedanib, inhibited TGFβ1-induced upregulation of Rcn3, suggesting pirfenidone would be more effective against the Rcn3-mediate loop. However, Rcn3 overexpression significantly repressed their effects against TGFβ1-induced fibroblast activation, suggesting the deregulation of Rcn3 expression may restrain the therapeutic effect of both pirfenidone and nintedanib. Therefore, the level of Rcn3 in IPF patients could be a valuable clinical prognostic marker or an index guiding IPF therapeutic strategies. Future prospective clinical studies are required to evaluate the clinical significance of Rcn3.

## Conclusions

Taken together, we uncover a determinate role of the TGFβ1-Rcn3-TGFBR1 loop in the pathogenesis of pulmonary fibrosis, as well as introduce a novel regulating mechanism of TGFβ1 signalling. These findings suggest that Rcn3 upregulation may cause resistance to IPF treatment and targeting Rcn3 could be a novel approach for pulmonary fibrosis treatment.

### Supplementary Information

**Additional file 1: Figure S1.** Rcn3 in lung fibroblasts is upregulated in the fibrotic lungs from either IPF patients or bleomycin-induced lung fibrosis mouse model. **Figure S2.** Mice with the selective disruption of Rcn3 in fibroblast developed normally and displayed normal inflammatory condition in the lung, but CKO mice exhibit alleviated fibrotic response to bleomycin instillation. **Figure S3.** CKO and control lungs showed comparable inflammatory response at 3, 7 and 14 days post bleomycin treatment. **Figure S4.** TGFβ1 treatment enhanced the transcriptions of *Rcn3* and fibrotic genes in lung fibroblast and Rcn3 knockdown significantly blunted the induction of fibrotic genes induced by TGFβ1 exposure rather than FGF exposure. **Figure S5.** qPCR analyses *of αSMA*, *Col1a1*, and *Col1a2* in human lung fibroblast with Rcn3 in response to pirfenidone or nintedanib treatment. **Figure S7.** The top 6 potential direct interaction models of EZH2-Rcn3 by protein–protein docking tool ClusPro serve. **Figure S8.** The immunofluorescence assay in Hela cells showed the cellular distribution of Rcn3 and co-localization with EZH2.

**Additional file 2: Table S1.** Primer sequences used in RT-qPCR. **Table S2.** Antibodies used in immunoblot. **Table S3.** Primer sequences used in ChIP RT-qPCR.

## Data Availability

All data generated or analyzed during this study are included in this published article and its Additional files.

## Abbreviations

IPF: Idiopathic pulmonary fibrosis
ILD: Intestinal lung diseases
Rcn3: Reticulocalbin 3
TGF β: Transforming growth factor β
FGF: Fibroblast growth factor
PDGF: Platelet-derived growth factor
TGFBR1/2: TGF β1 type I/II receptor
ER: Endoplasmic reticulum
UPR: Unfolded protein response
AEC II: Type II alveolar epithelial cells
ALI: Acute lung injury
DHLF: Disease human lung fibroblast
NHLF: Normal human lung fibroblast
MLF: Mouse lung fibroblast
H3K27me3: Tri-methylation of histone H3 at Lys27
qPCR: Quantitative real-time PCR
CoIP: Co-immunoprecipitation
ChIP: Chromatin immunoprecipitation
EDU: 5-Ethynyl-2-deoxyuridine
FVC: Forced vital capacity
Re: Expiratory resistance
Ri: Inspiratory resistance
Cdyn: Dynamic compliance
CCND1: Cyclin D1
PCNA: Proliferating cell nuclear antigen
4-PBA: 4-Phenylbutyrate
CCK8: Cell Counting Kit-8
Col1a1/2: Collagen I type 1/II
α-SMA: Actin alpha 2
PRC2: Poly comb repressive complex 2
EZH2: Epigenetic methyltransferase in poly comb repressive complex 2
rhEZH2: Recombinant human EZH2 protein
