# Supplementary material for: TGFβ1-RCN3-TGFBR1 loop facilitates pulmonary fibrosis by orchestrating fibroblast activation
Source: Respir Res. 2023 Sep 14;24:222. doi: 10.1186/s12931-023-02533-z (PMC10500825; doi:10.1186/s12931-023-02533-z)
Supplement: Supplementary file 1 — Additional file 1: Figure S1. Rcn3 in lung fibroblasts is upregulated in the fibrotic lungs from either IPF patients or bleomycin-induced lung fibrosis mouse model. Figure S2. Mice with the selective disruption of Rcn3 in fibroblast developed normally and displayed normal inflammatory condition in the lung, but CKO mice exhibit alleviated fibrotic response to bleomycin instillation. Figure S3. CKO and control lungs showed comparable inflammatory response at 3, 7 and 14 days post bleomycin treatment. Figure S4. TGFβ1 treatment enhanced the transcriptions of Rcn3 and fibrotic genes in lung fibroblast and Rcn3 knockdown significantly blunted the induction of fibrotic genes induced by TGFβ1 exposure rather than FGF exposure. Figure S5. qPCR analyses of αSMA, Col1a1, and Col1a2 in human lung fibroblast with Rcn3 in response to pirfenidone or nintedanib treatment. Figure S7. The top 6 potential direct interaction models of EZH2-Rcn3 by protein–protein docking tool ClusPro serve. Figure S8. The immunofluorescence assay in Hela cells showed the cellular distribution of Rcn3 and co-localization with EZH2. [file 12931_2023_2533_MOESM1_ESM.pdf]

Fig S1 Rcn3 in lung fibroblasts is upregulated in the fibrotic lungs from either IPF patients or bleomycin-induced lung fibrosis mouse model

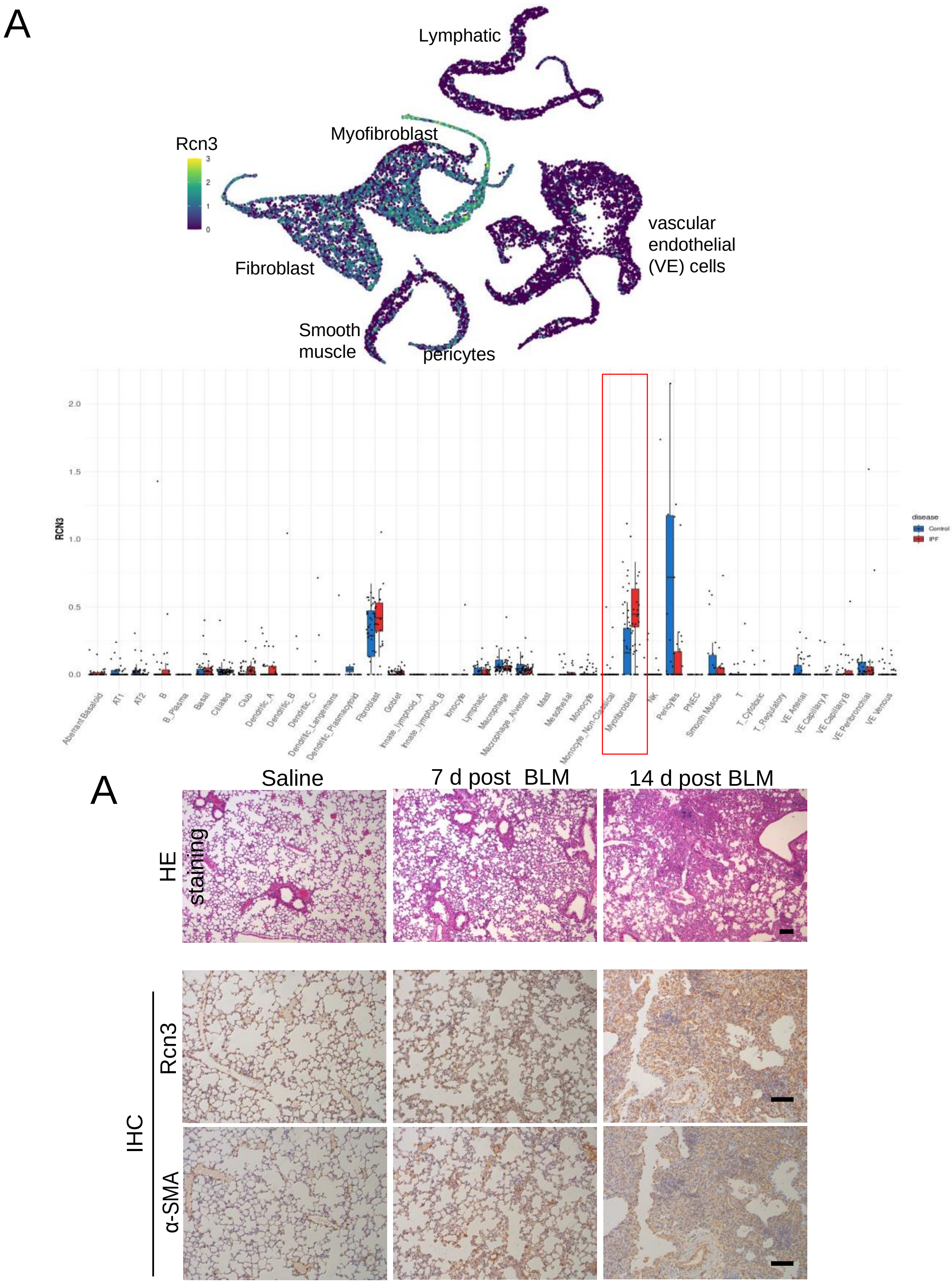

Fig S1 Rcn3 in lung fibroblasts was upregulated in the fibrotic lungs induced by intratracheal bleomycin instillation. **(A)** The Single-cell RNA-seq on IPF patients revealed a remarkable upregulation of Rcn3 in myofibroblasts. The Rcn3 expression is analyzed through the web tool: www.ipfcellatlas.com based on published data: *Sci. Adv.* **6**, eaba1983 (2020). **(B)** The HE staining of lung sections from C57BL/6J mice at 7 and 14 days post intratracheal bleomycin instillation. immunohistochemistry (IHC) staining on the normal lung sections and fibrotic continues lung sections using anti-α-SMA and anti-Rcn3 antibodies; scale bar: 100 μm.

Fig S2 Mice with the selective disruption of Rcn3 in fibroblast developed normally and displayed normal inflammatory condition in the lung, but CKO mice exhibit alleviated fibrotic response to bleomycin instillation.

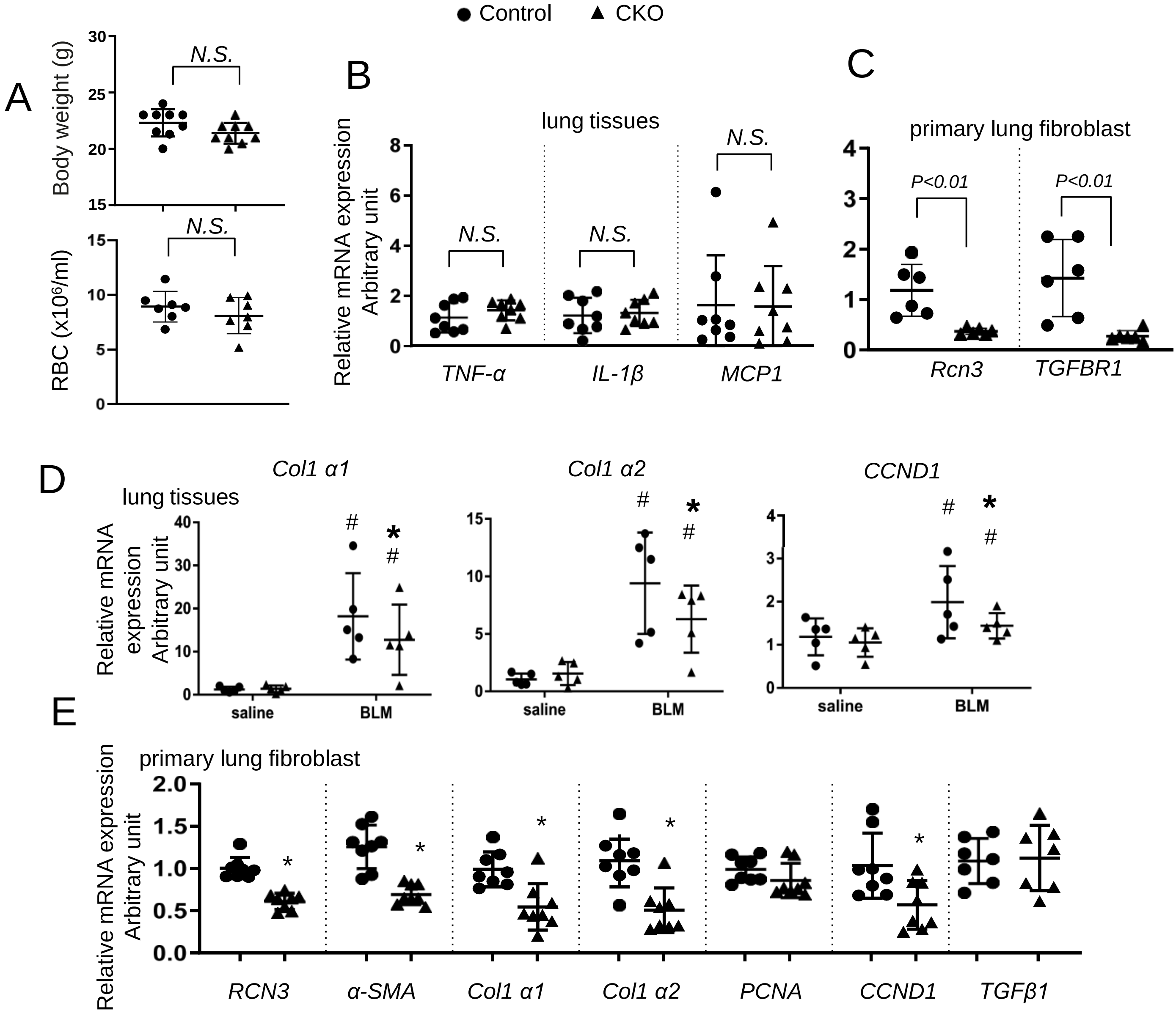

Fig S2 Mice with the selective disruption of Rcn3 in fibroblast develop normally and display normal inflammatory condition in the lung, but CKO mice exhibit alleviated fibrotic response to bleomycin instillation. **(A)** CKO mice show normal bodyweight and comparable red blood cell (RBC) count in adulthood (2 months old, n=7-9). **(B)** The quantitative PCR (qPCR) examined the level of representative inflammatory cytokines in the lung (n = 8). **(C)** The mRNA levels of Rcn3 and TGFBR1 in the primary lung fibroblast from control and CKO mice (n= 6). **(D)** The fibrotic genes in the lung tissues were analyzed by qPCR at 14 days after intratracheal bleomycin instillation (n = 5). **(E)** The lung fibroblast from CKO mice exhibited significantly depressed inductions of fibrotic genes post bleomycin treatment (n = 8). The qPCR data were normalized to the RPL19 content and analyzed by the $2^{-\Delta\Delta Ct}$ method relative to saline-treated control group. Data are presented as the mean ± SD with statistical analysis performed by unpaired student's, Mann-Whitney U-tests or two-way ANOVA (Tukey post hoc test) as appropriate. # p<0.05 vs vehicle treatment at same siRNA group; * p<0.05 vs Ctl-siRNA at same treatment. t. CKO: conditional knock; TGFBR1: TGFβ receptor 1; BLM: bleomycin; Col1 a1/2: collagen I a1/2; PCNA: Proliferating cell nuclear antigen; CCND1: Cyclin D1.

Fig S3 CKO and control lungs showed comparable inflammatory response at 3, 7 and 14 days post bleomycin treatment

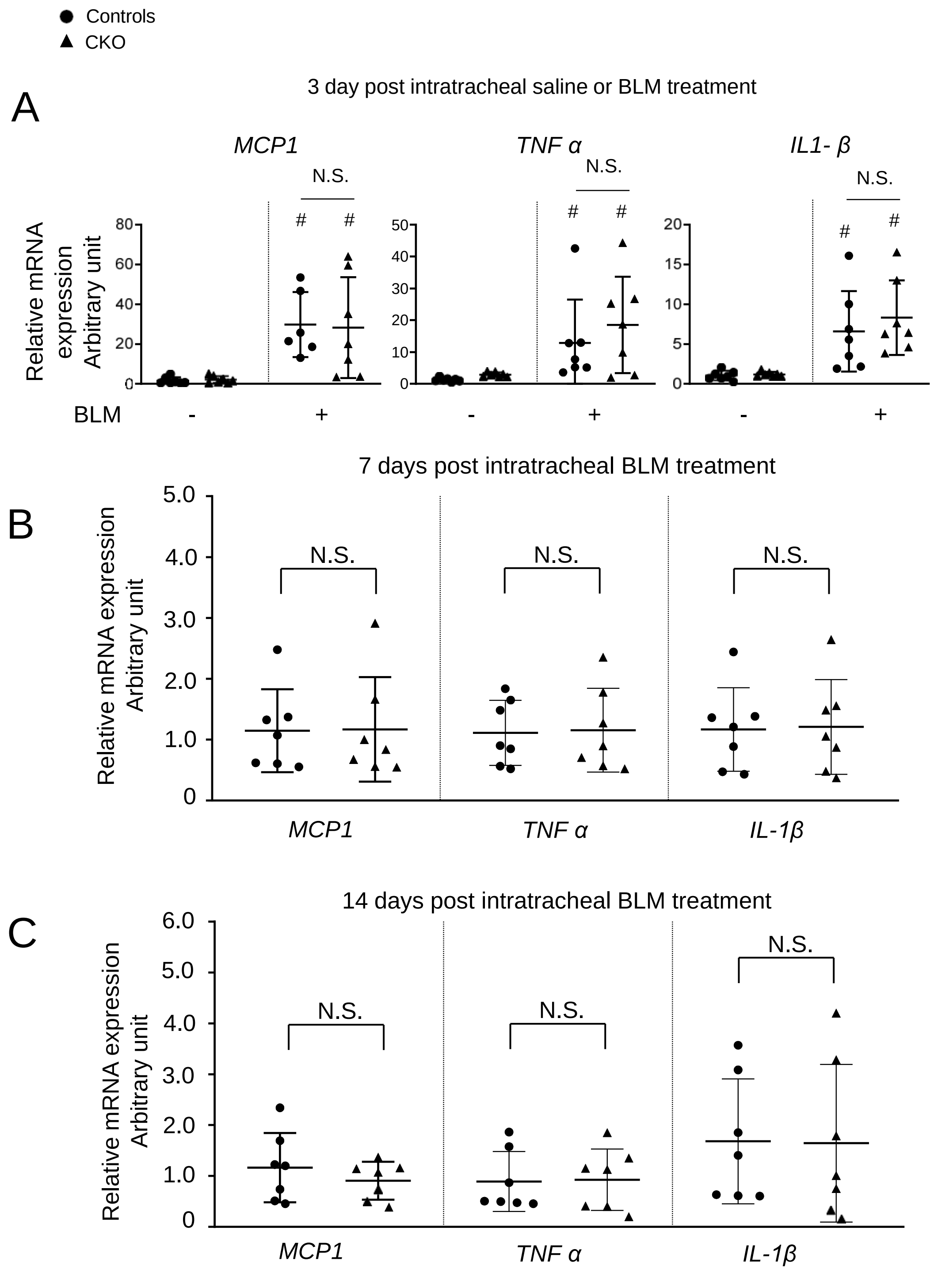

Fig S3 CKO and control lungs showed comparable inflammatory at 3, 7 and 14 days post bleomycin treatment. qPCR analyses of the mRNA expressions of proinflammatory cytokines in the lung at 3 (A) , 7 days (B) and 14 day (C) after intratracheal bleomycin instillation (0.08 U/kg). The qPCR data were normalized to the RPL19 content and analyzed by the $2^{-\Delta\Delta Ct}$ method relative to BLM-treated control group. Data presented as mean ± SD; n = 7 per group; # $p<0.05$ versus saline controls. NS: not significant different between two groups. Control: littermate controls; CKO: conditional knock; BLM: bleomycin.

Fig S4 TGFβ1 treatment enhanced the transcriptions of *Rcn3* and fibrotic genes in lung fibroblast and Rcn3 knockdown significantly blunted the induction of fibrotic genes induced by TGFβ1 exposure rather than FGF exposure.

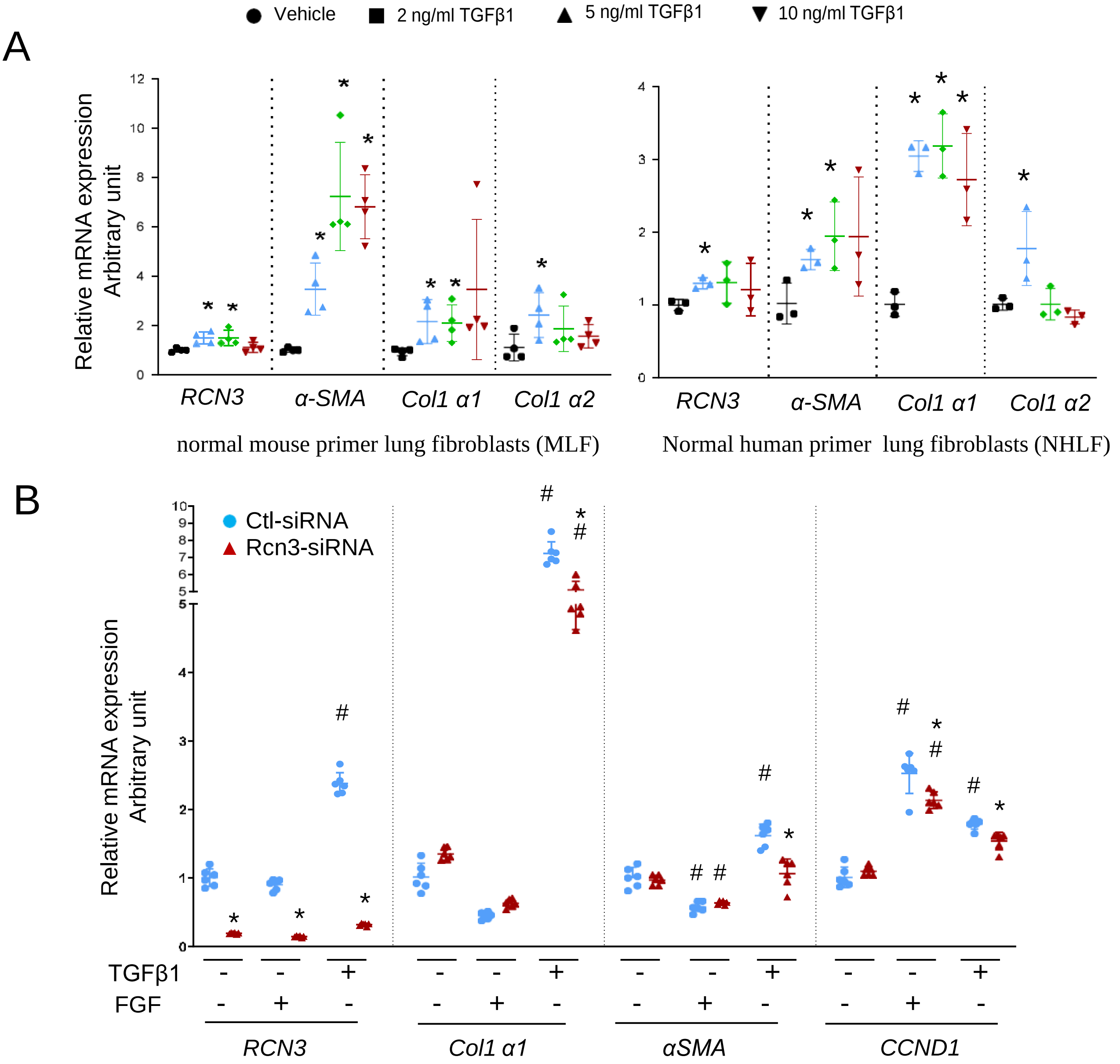

Fig S4 TGFβ1 treatment enhanced the transcriptions of *Rcn3* and fibrotic genes in lung fibroblast and Rcn3 knockdown significantly blunted the induction of fibrotic genes induced by TGFβ1 exposure rather than FGF exposure. **(A)** TGFβ1 treatment enhanced the transcriptions of *Rcn3* and fibrotic genes in mouse primer normal lung fibroblasts (MLF) and normal human lung fibroblasts (NHLF). LF were treated by TGFβ1 at different concentrations for 24 hours; the mRNA levels of *Rcn3, α-SMA, Col1 a1* and *Col1 a2* were analyzed by qPCR (n=3-4). The data were normalized to *RPL19* (MLF) or *GAPDH* (NHLF) content and analyzed by the $2^{-\Delta\Delta Ct}$ method relative to vehicle control group. Data presented as mean ± SD; two-tailed Student's t-test or the Mann–Whitney U test for statistical comparisons between groups. **(B)** NHLF transfected by Ctl-siRNA or Rcn3-siRNA were subjected to TGFβ1 (5 ng/ml) or FGF (50 ng/ml) exposure for 24 hours and the mRNA levels *of Rcn3, αSMA, Col1a1* and *CCND1* were analyzed by qPCR (n = 6). The data were normalized to the GAPDH content and analyzed by the $2^{-\Delta\Delta Ct}$ method relative to vehicle Ctl-siRNA group. Data presented as mean ±SD; 2-way ANOVA (Tukey post hoc test). # $p<0.05$ vs vehicle treatment at same siRNA group; * $p<0.05$ vs Ctl-siRNA at same treatment. Ctl-siRNA: control-siRNA; Col1 a1: collagen1 a1; CCND1: Cyclin D1.

Fig S5 qPCR analyses *of αSMA, Col1a1,* and *Col1a2* in human lung fibroblast with Rcn3 in response to pirfenidone or nintedanib treatment.

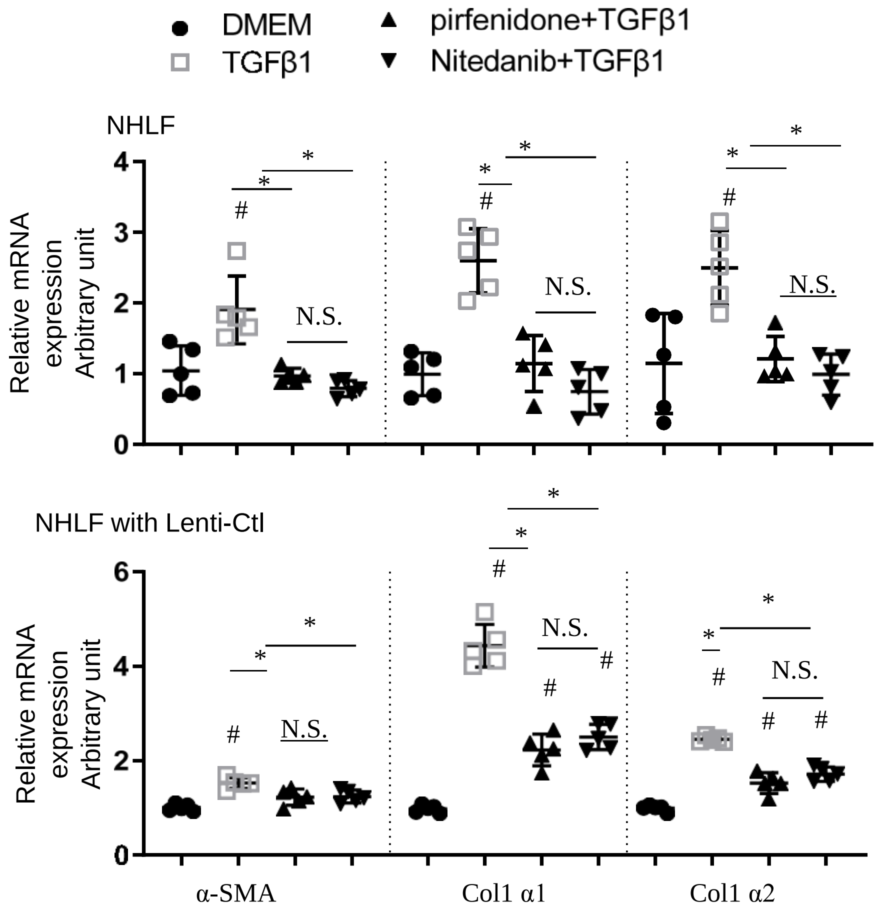

Fig S5 qPCR analyses of *α-SMA*, *Col1a1*, and *Col1a2* in normal human lung fibroblast (NHLF) and NHLF with control lentinoviur lenti-control in response to pirfenidone or nintedanib. NHLF infected by lentivirus-control (lenti-Ctl) were subjected to co-treatment of TGF-β1 with pirfenidone at 100 ng/ml (540 nM) or nintedanib at 150 ng/ml (278 nM) for 24 hours and the mRNA levels of *Rcn3, α-SMA, Col1a1 and Col1a2* were analyzed by qPCR. Both pirfenidone and nintedanib suppressed TGF-β1-induced activation of NHLF; NHLF infected with lenti-Ctl showed similar results to the wildtype NHLF. The data were normalized to the GAPDH content and analyzed by the $2^{-\Delta\Delta Ct}$ method relative to vehicle Ctl-siRNA group. Data presented as mean ±SD; n = 6 per group; 1-way ANOVA (Tukey post hoc test) . # $p<0.05$ vs vehicle controls; * $p<0.05$ as indicated. Lenti-Ctl: lentivirus-control; Lenti-Rcn3: lentivirus-Rcn3; Lenti-Rcn3+P: lentivirus-Rcn3+pirfenidone; Lenti-Rcn3+N: lentivirus-Rcn3+ nintedanibe; Col1a1: collagen1 a1; Col1a2: collagen1 a2

Fig S6 Rcn3 deficiency significant depressed the expression of TGFBR1 but not TGFBR2

A

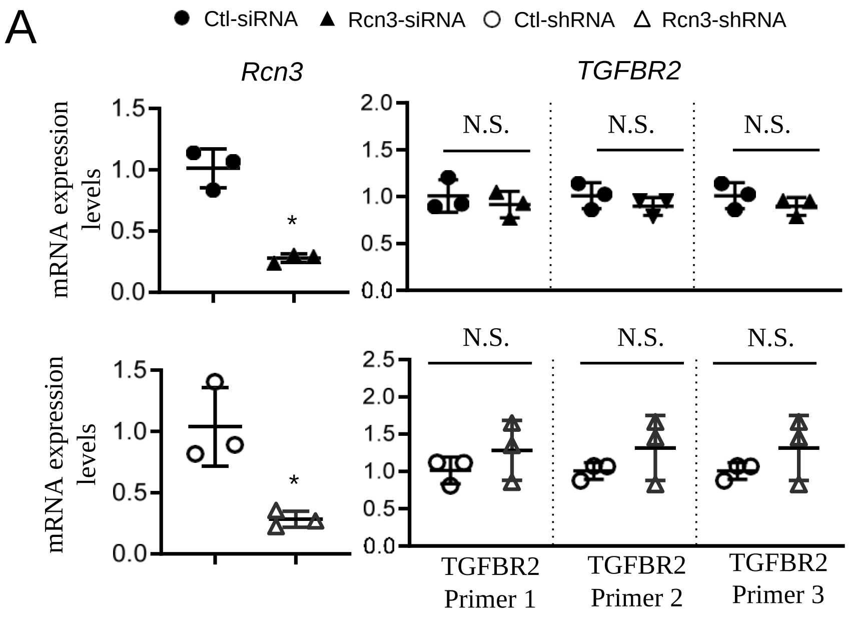

hTGFBR2	location	sequence
Primer 1	F: 432-452	CTGCCCATCCACT GAGACATA
	R: 675-656	AGCTTGGGGTCA TGGCAAAC
Primer 2	F: 799-821	GTAGCTCTGATG AGTGCAATGAC
	R: 930-910	CAGATATGGCAA CTCCCAGTG
Primer3	F: 431-451	ACTGCCCATCCAC TGAGACAT
	R: 622-601	CCATACAGCCAC ACAGACTTCC

B

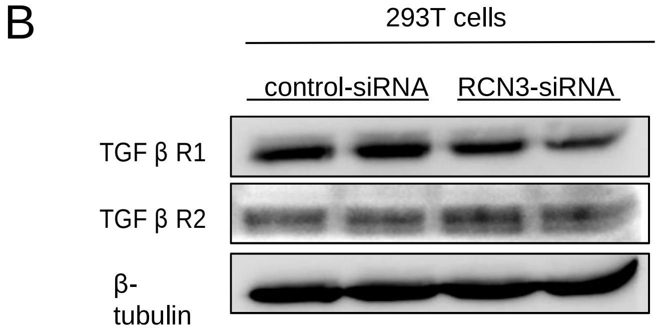

Fig S6 Rcn3 deficiency significant depressed the expression of TGFBR1 but not TGFBR2. **(A)** qPCR analyses of *Rcn3* and *TGFBR2* in human lung fibroblast with Rcn3 knockdown through siRNA or shRNA methods. Three primer sets were used to examine *TGFBR2* mRNA level in these cells. The data were normalized to the GAPDH content and analyzed by the $2^{-\Delta\Delta Ct}$ method relative to control group. Data presented as mean ±SD; n = 3 per group * $p<0.05$ versus controls. **(B)** The immunoblot assay showed the marked decrease of TGFBR1 expression in 293T cells transfected with Rcn3-siRNA, in which the expression of TGFBR2 was not altered. The β-tubulin expression is as loading control.

Fig S7. The top 6 potential direct interaction models of EZH2-Rcn3 by protein-protein docking tool ClusPro serve.

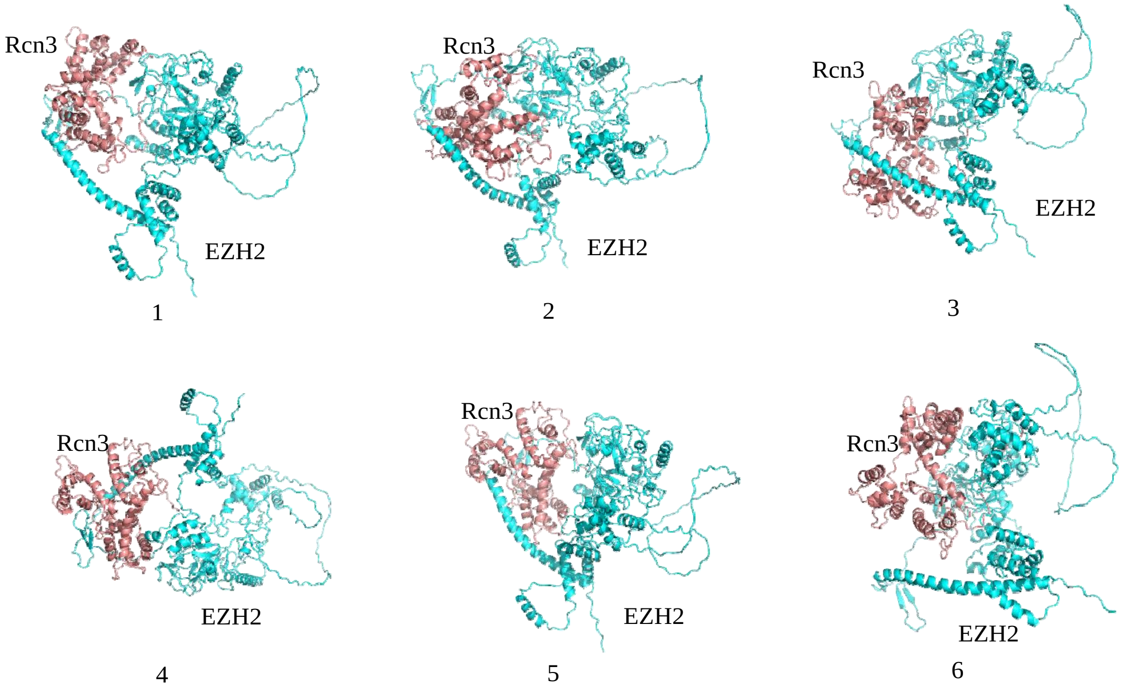

Cluster	Members	Representative	Balanced Weighted Score
1	47	Center	-1360.1
		Lowest Energy	-1503.9
2	43	Center	-1282.6
		Lowest Energy	-1614.8
3	38	Center	-1227.5
		Lowest Energy	-1503.6
4	30	Center	-1228.9
		Lowest Energy	-1558.1
5	26	Center	-1287.2
		Lowest Energy	-1425.4
6	23	Center	-1235.4
		Lowest Energy	-1383.8

Fig S7. The top 6 potential direct interaction models of EZH2-Rcn3 by protein-protein docking tool ClusPro serve. The predicted structures of EZH2 and Rcn3 obtain from the UniProt by AlphaFold model (AF-Q15910 and AF-Q96D15) were subjected into ClusPro web-based server to analyze their interaction (http://cluspro.bu.edu/). The results suggested a potential direct interaction between EZH2 and Rcn3 with reasonable balanced weighted scores. The The top 6 potential direct interaction models of showed Rcn3 could directly dock into EZH2 protein. EZH2 model is represented in cyan colored ribbon diagrams and Rcn3 models represented in salmon colored ribbon diagrams in the different models. The docking scores for the top 6 models were showed as a table at the bottom, including the interaction segment members and balanced weight scores.

Fig S8 The immunofluorescence assay in Hela cells showed the cellular distribution of Rcn3 and co-localization with EZH2.

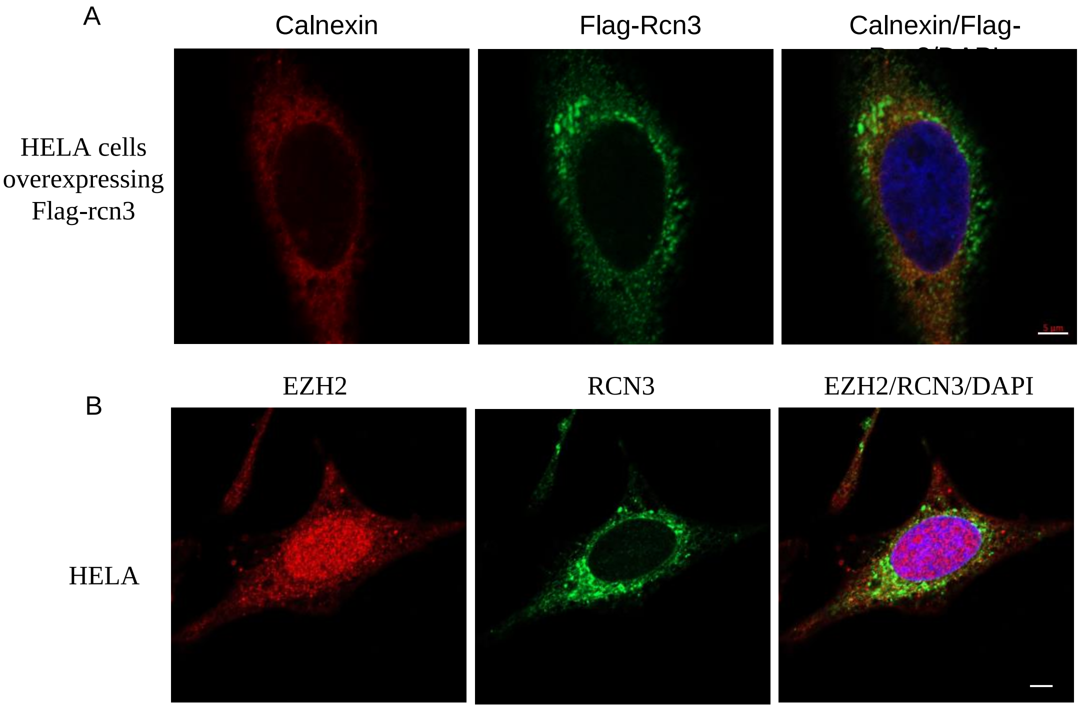

Fig S8 The immunofluorescence assay in Hela cells showed the cellular distribution of Rcn3 and co-localization with EZH2. **(A)** The immunofluorescence staining on the cells overexpressing Flag-Rcn3 using anti-Calnexin (ER marker, red) and anti-flag (green) antibodies and DAPI was for nuclear staining. The merged view showed both ER (yellow) and cytoplasmatic (green) localizations of Rcn3. **(B)** The immunofluorescence staining of EZH2 (red) and Rcn3 (green), The merged view showed (yellow) co-localization of Rcn3 with EZH2. Scale bar: 5 µm
