# Supplementary material for: TGFβ1-RCN3-TGFBR1 loop facilitates pulmonary fibrosis by orchestrating fibroblast activation
Source: Respir Res. 2023 Sep 14;24:222. doi: 10.1186/s12931-023-02533-z (PMC10500825; doi:10.1186/s12931-023-02533-z)
Supplement: Supplementary file 2 — Additional file 2: Table S1. Primer sequences used in RT-qPCR. Table S2. Antibodies used in immunoblot. Table S3. Primer sequences used in ChIP RT-qPCR. [file 12931_2023_2533_MOESM2_ESM.docx]

**Table S1**. Primer sequences used in RT-qPCR

| Primers for human genes | |
| --- | --- |
| hGAPDH-F | GAAGGTGAAGGTCGGAGTC |
| hGAPDH-R | GAAGATGGTGATGGGATTTC |
| hCollagenI α1 F | GAGGGCCAAGACGAAGACATC |
| hCollagenI α1 R | CAGATCACGTCATCGCACAAC |
| hCollagenI α2 F | GTTGCTGCTTGCAGTAACCTT |
| hCollagenI α2 R | AGGGCCAAGTCCAACTCCTT |
| hα-SMA F | GGCATTCACGAGACCACCTAC |
| hα-SMA R | CGACATGACGTTGTTGGCATAC |
| hPCNA F | CCTGCTGGGATATTAGCTCCA |
| hPCNA R | CAGCGGTAGGTGTCGAAGC |
| hCCND1 F1 | TGGAGCCCGTGAAAAAGAGC |
| hCCND1 R1 | TCTCCTTCATCTTAGAGGCCAC |
| hRcn3-F | CATGCGGGACATCGTGATTG |
| hRcn3-R | CTCGGCTGAGTACAGATCCG |
| hTGFBR1 F1 | GACAACGTCAGGTTCTGGCTCA |
| hTGFBR1 R1 | CCGCCACTTTCCTCTCCAAACT |
| hTGFBR2 primer 1 F | CTGCCCATCCACTGAGACATA |
| hTGFBR2 primer 1 R | AGCTTGGGGTCATGGCAAAC |
| hTGFBR2 primer 2 F | AGCTTGGGGTCATGGCAAAC |
| hTGFBR2 primer 2 R | GTAGCTCTGATGAGTGCAATGAC |
| hTGFBR2 primer 3 F | CAGATATGGCAACTCCCAGTG |
| hTGFBR2 primer 3 R | ACTGCCCATCCACTGAGACAT |
| hEZH2 F | CCATACAGCCACACAGACTTCC |
| hEZH2 R | ATCCCCGTGTACTTTCCCATCATAAT |
| Primers for mouse genes | |
| IL1 beta F | GAAATGCCACCTTTTGACAGTG |
| IL1 beta R | TGGATGCTCTCATCAGGACAG |
| TNF alpha F | CAGGCGGTGCCTATGTCTC |
| TNF alpha R | CGATCACCCCGAAGTTCAGTAG |
| MCP-1 F | TAAAAACCTGGATCGGAACCAAA |
| MCP-1 R | GCATTAGCTTCAGATTTACGGGT |
| Col1a1 F | CCAAGAAGACATCCCTGAAGTCA |
| Col1a1 R | TGCACGTCATCGCACACA |
| Col1a2 F | AGCTTTGTGGATACGCGGACT |
| Col1a2 R | TCGTACTGATCCCGATTGCA |
| α-SMA F | CGGGAGAAAATGACCCAGATT |
| α-SMA R | GGACAGCACAGCCTGAATAGC |
| RCN3 F | GGGAATTTCCAGTACGACCA |
| RCN3 R | AAGATCCATGCGGTCTACGA |
| RPL19 F | ATGAGTATGCTCAGGCTACAGA |
| RPL19 R | GCATTGGCGATTTCATTGGTC |
| Cycling D1 F | TCACCCTGAGAGTAGGGAGC |
| Cycling D1 R | GGCCTTCAGGCAAAAACCAG |
|  |  |
|  |  |

**Table S2**. Antibodies used in immunoblot

| Antibody | Brand | Catalog N.O. |
| --- | --- | --- |
| RCN3 | Sigma | HPA050402 |
| collagen I | Abcam | ab260043/ab21286 |
| α-SMA | abcam | ab124964 |
| BioID | abcam | ab232733 |
| GAPDH | Yeasen | 30201ES60 |
| β-tubulin | Yeasen | 30301ES60 |
| GRP78 | Santa Cruz | sc13968 |
| p-stat3 | CST | 9145s |
| stat3 | Santa Cruz | sc-8019-SNT |
| p-AKT | CST | 4060s |
| AKT | Santa Cruz | sc5298 |
| p-smad3 | abcam | ab52903 |
| smad3 | abcam | ab208182 |
| TGFBR1 | abcam | ab1235578 |
| TGFBR2 | abcam | ab259360 |
| Na-K ATPase | CST | 23565s |
| EZH2 | CST | 5246s |
| H3K27me3 | CST | 9733s |
| Histone 3 | CST | 4499T |
| Flag | sigma | F1804-50ug |
| Rabbit IgG | Beyotime | A7028 |
| mouse IgG | Beyotime | A7016 |
| EZH2 | Santa Cruz | sc-13725s |

**Table S3**. Primer sequences used in ChIP RT-qPCR

| TGFBR1 R1 | F | 5’-actggaactgagagggcaaa-3’ |
| --- | --- | --- |
|  | R | 5’-caaactcccctcttgctcag-3’ |
| TGFBR1 R2 | F | 5’-agctgcggtgtagagcaagt-3’ |
|  | R | 5’-gaaaaggcgtggatattgga-3’ |
| TGFBR1 R3 | F | 5’-atcctggatctgtgctggag-3’ |
|  | R | 5’-ccaaacccagaaagtcctca-3’ |
| TGFBR1 R4 | F | 5’-cgcgcctagaggaggttag-3’ |
|  | R | 5’-tagagcgatgggtgtgtctg-3’ |
